# Shaking into deficits: investigating behavioural and neuropathological outcomes associated with a novel preclinical model of infant abusive head trauma

**DOI:** 10.1186/s40478-025-02029-5

**Published:** 2025-05-15

**Authors:** Sydney A. Harris, Marissa Sgro, Sabrina Salberg, Crystal Li, Elaina Vlassopoulos, Madeleine Smith, Bridgette D. Semple, Holly R. Chinnery, Richelle Mychasiuk

**Affiliations:** 1 Department of Neuroscience, School of Translational Medicine, Monash University, Melbourne, Australia; 2 Department of Optometry and Vision Science, University of Western Australia, Lions Eye Institute, Perth, Western Australia Australia; 3 Department of Neuroscience, School of Translational Medicine, Monash University, The Alfred Centre, 99 Commercial Road, Melbourne, VIC 3004 Australia

**Keywords:** Pediatric brain injury, Postnatal development, White matter, Gene expression, Traumatic brain injury, Neuroinflammation, Blood-brain-barrier integrity

## Abstract

Abusive head trauma (AHT) resulting from violent shaking and whiplash-induced brain injury by a caregiver, is the leading cause of abusive mortality and morbidity in children. Cerebral oedema is common in survivors of AHT. While many children may initially appear behaviourally asymptomatic or present with non-specific symptoms following the AHT, deficits often emerge later in childhood. Additionally, AHTs are frequently repetitive, with a single child likely to experience multiple AHTs. Despite the prevalence of AHT, the mechanisms that lead to brain pathology and the latent emergence of behavioural deficits are poorly understood, and there is a paucity of preclinical, small animal models to investigate the biology and cumulative effects of repetitive injuries. This study aimed to develop a preclinical model of repetitive AHT and subsequently examine alterations in gene expression, cell types, and early adolescent behaviour. Mice were placed on a 400 rpm shaking device for 60s. This was repeated one, three, or five times throughout the neonatal development period (postnatal days (P)8–12). Injured mice initially displayed no overt behavioural changes compared to uninjured controls; however, in adolescence (P40-45) they later developed deficits in socialisation and thermal nociception. Further, alterations in the expression of genes involved in growth, cell damage, and development were observed in the brains of injured mice, along with an increase in white matter cells and evidence of blood-brain barrier leakage. This novel preclinical model of AHT provides a valuable platform for exploring diagnostic biomarkers and potential therapeutic interventions for children with an AHT.

**Supplementary Information:**

The online version contains supplementary material available at 10.1186/s40478-025-02029-5.

## Introduction

Traumatic brain injuries (TBIs) are a major cause of death and disability in children worldwide [17, 18]. Approximately one-third of children under 3 years of age admitted to hospitals with head injuries experienced a TBI that was abusive in nature and inflicted by a trusted caregiver [83, 84, 92]. Abusive head trauma (AHT; formerly referred to as shaken baby syndrome) is the leading cause of abusive mortality and morbidity in children under two [48, 79]. This injury occurs most commonly when a caregiver tightly grasps a child’s body and vigorously shakes them, overwhelming the neck muscles, which results in acceleration/deceleration forces that cause the developing brain to move within the skull [55, 67, 79]. These injuries are most commonly induced when a child is between three to eight months of age; a time period characterised by high water content in the brain and the development of white matter tracts and synaptic connections, which leaves the brain highly susceptible to injury [81].

Accurate diagnosis of an AHT in the hospital setting is difficult, as physical trauma only presents in one-third of cases; and even when symptoms are initially present, they are generally non-specific [7, 35, 75, 77]. Additionally, many symptoms such as behavioural disabilities, social deficits, and brain abnormalities do not manifest until later in childhood [5, 6, 12, 13, 27, 49, 66]. This often leads to children being discharged back into abusive settings where they are exposed to repetitive AHTs. Indeed, an estimated 31–55% of AHTs are repetitive with reports indicating that a single child may sustain up to 30 shaking incidences before diagnosis or death [2, 28, 65]. Similar to repetitive mild TBIs (i.e., concussions), which were historically considered benign but are increasingly appreciated for their cumulative negative consequences, repetitive AHTs, even those lacking initial symptom presentation, may lead to potentiated damage and long-term deficits.

AHTs that do not result in mortality are devastating for survivors, with a myriad of lifelong consequences. Preclinical animal models allow for the investigation of the pathophysiological processes behind these deficits that are not feasible or ethical to examine in human populations. There are currently no animal models of AHT that accurately represent the injuries and deficits seen in human children that experience an AHT. There is also no consensus regarding the best way to model AHT, which is further complicated by the fact that many of the current animal models focus on a single severe AHT, that leads to death in a proportion of the cohort. A robust preclinical model should account for the development of pathologies that do not manifest until later in life, while also incorporating the progression of damage that occurs as injuries become repetitive [37]. Therefore, the purpose of this study was to generate and validate a developmentally relevant mouse model of AHT that replicates the known pathophysiological changes that manifest in human children exposed to repetitive AHT in infancy. Building upon prior studies [11, 12], we developed a mouse model of repetitive AHTs that leads to brain oedema, changes in oligodendrocyte cell quantities, and modifications to gene expression in the prefrontal cortex and hippocampus, as well as the protracted development of behavioural deficits. Ultimately, this mouse model is the first to demonstrate the progression of deficits from single to repetitive AHTs and permits the investigation of deficits that manifest later in life.

## Methods

### Animals

All research was completed in compliance with the Precinct Animal Centre (PAC) Animal Care Guidelines at Monash University. Ethics approval was granted by the Alfred Medical Research and Education Precinct Animal Ethics Committee. All experiments were completed in accordance with the approved ethical stipulations as outlined in E/8303/2022/M. C57Bl/6J female and male adult mice were obtained from the Monash Animal Research Platform. Mice were kept within the PAC facility and maintained on a 12 h/12hr light/dark cycle (lights on at 0700) with *ad libitum* access to food and water. When mice were approximately 10 weeks old, they were bred in a harem whereby two females were paired with one male for five days. Plugs confirmed pregnancy and dam weight was monitored for the duration of pregnancy. Dams were housed in groups of four after mating until embryonic day (E) 18, at which point they were singly housed. Day of parturition was considered postnatal day (P) 0. All experimental procedures were allowed variance of ± 1 day, to compensate for slight differences in the date of birth. Entire litters were used, with offspring of both sexes allocated across experimental groups to avoid possible confounding effects associated with any specific litter. Mice were sexed on P7. Dams were singly housed with their offspring until offspring were P21, at which time the offspring were removed from their dam and housed in same-sex groups of 4–6 mice. Only primiparous dams were used. 

### AHT induction

#### 15s injury

**Fig. 1:**
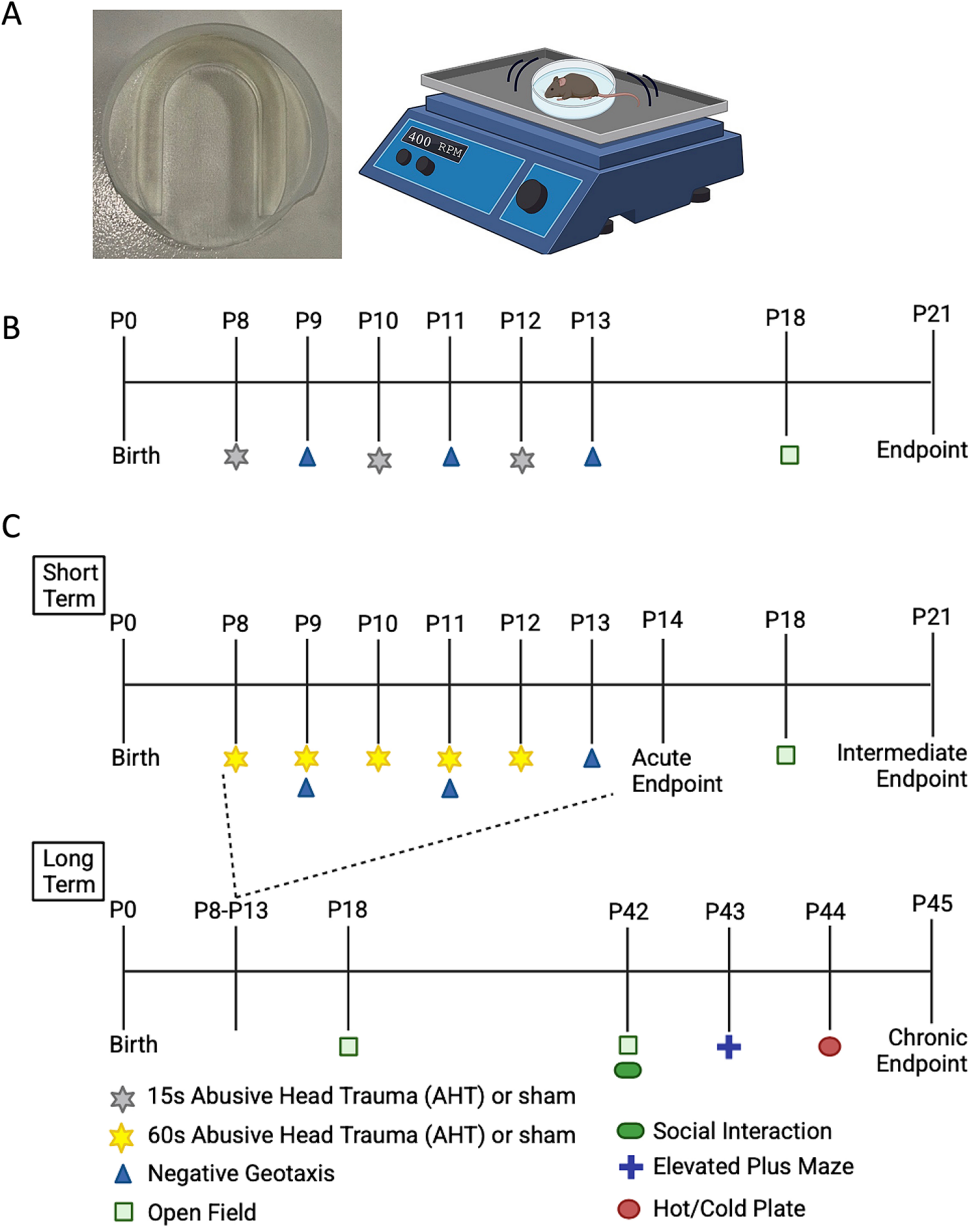
(**A**) Custom 3D printed mouse holder for AHT administration and illustrative representation of the shaking device. (**B**) Timeline used in the 15s injury paradigm. (**C**) Short term and long-term timeline of the 60s injury paradigm

On P8-P12 mice received a shaking injury, to model an AHT. Individual mice were loosely wrapped in 5 cm x 5 cm piece of cotton and secured into to a custom 3D printed holder (Fig. 1A) using Blu Tack^®^. Care was taken to ensure that the head and neck of the mouse could move freely and was not secured in place. Mice were then placed on a 400 rpm shaking device maintained at 25^o^C (Medium orbital shaking incubator, model OM15, Ratek Instruments Pty Ltd, Australia). Mice were placed on the device for 15 s either (a) once on P8, or (b) three times, once daily on P8, P10, and P12. Sham injuries consisted of similarly securing the mouse to the holder and placing it on the shaker, while stationary, for 15s. After the 15s injury or sham procedure, mice were immediately removed from the device and were monitored for breathing and movement. Mice were placed back with their littermates on a heating pad until the entire litter had received an injury or sham, at which time the litter was returned to the dam and home cage. There was no observed apnoea or manual resuscitation required following this injury. Mice from this cohort performed an open field task (described below) on P18 and were euthanised for tissue collection on P21. In total, 120 mice were used in this protocol with roughly equal females and males (sham *n* = 39, 1 injury *n* = 40, 3 injury *n* = 41). Individual data points within the figures represents individual animals. An experimental timeline can be found in Fig. 1B.

#### 60s injury

The severity of the AHT was increased from the 15s injury described above in a separate cohort of mice by increasing the number, frequency, and duration of the injury. In this paradigm, mice received a 60s shaking injury at 400 rpm using the same device and methods described above. Mice were subjected to either (a) a single injury on P8 or P12; (b) three injuries, once daily on P8, P10, and P12; or (c) five injuries occurring once daily from P8-P12. As described above, another group of mice received only sham injuries, and mice that were in the single injury and three injury groups received the sham procedure on the days where they did not have a shaking injury. Using this injury paradigm, mice were humanely euthanised at P14, P21, and P45, to examine acute, intermediate, and chronic brain and behavioural changes (Fig. 1C). For this procedure, a total of 272 offspring were used with approximately equal females and males (sham *n* = 61, 1 injury *n* = 72, 3 injury *n* = 67, 5 injury *n* = 72). Individual data points within the figures represents individual animals. Apnoea was observed following 5.2% of injuries and manual resuscitation was required following 1.3% of injuries. No mice were apnoeic following a sham procedure. One mouse (0.15%) died following a single injury on P8; however, there was no additional mortality.

### Behavioural testing

Mice were subjected to an acute battery of behavioural tests between P8-P18, and a chronic battery from P42-P44. All behavioural tasks were performed under indirect white light (480 lx), unless otherwise stated and mice were habituated to the testing room in their home cage for 30 min prior to testing. All behavioural testing occurred between 0900 and 1500 h by an investigator masked to the experimental conditions. Apparatuses were cleaned with 80% ethanol between mice, unless otherwise stated.

#### Negative geotaxis

Negative geotaxis is used to assess developmental reflexes in young mice, specifically proprioception [71, 98]. For this task, mice were placed on a 45-degree inclined plane with their head facing downwards. The time it took for the mouse to turn its head, beyond the horizontal plane, and face upwards, was scored. Mice performed this task on P9, P11, and P13, 30 min after their respective injuries. Mice across all cohorts performed this task.

#### Forelimb Grasp

The ability for a young mouse to grasp a bar and hang develops at approximately P8 and is an indicator of strength [40]. For this task, mice were positioned with their forepaws resting on a small, horizontal, metal rod that was 10 cm above bedding, until they grasped the rod. The mouse was then released and the time that the mouse was able to hang was recorded. This was repeated until the mouse could hang for more than one second for two consecutive days. If the mouse immediately fell without grasping, the test was repeated once. This test was performed on mice in the 60s injury paradigm, daily from P8 until they reached criteria.

#### Air righting reflex

The ability for a mouse to right themselves into a prone position in the air develops at approximately P11 and indicates coordination and proprioception [40]. For this task, mice were suspended in supine position 10 cm over bedding. Mice were released and the position they landed in was observed and scored. This test was repeated once daily from P8 until mice were able to land on all four paws for two consecutive days. This was performed approximately one hour post injury in mice in the 60s injury cohort.

#### Open field task

On P18 and P42 mice in the intermediate (P21 euthanasia) and chronic (P45 euthanasia) groups performed an open field task, as previously described [87]. In brief, mice were placed in the centre of a square arena (40 cm W x 40 cm L x 30 cm H) and were allowed to freely explore the empty arena for five minutes. Time, velocity, and distance travelled was automatically recorded and analysed using TopScan Lite Software (Clever Sys Inc., Reston, VA). The video analysis software divided the arena into a centre zone, comprised of the central 50% of the arena (20 cm x 20 cm central square), and an outside zone, that consisted of the outer 50% of the arena. Activity in the centre zone and outside zone were analysed separately.

#### Social interaction

On P42 mice in the chronic group performed a same-sex socialisation task [89]. Stimulus mice were naïve, age, and sex-matched C57Bl/6J mice that were group housed separately from the test mice. On test day, mice were first allowed to habituate to a 30 cm x 30 cm empty arena for five minutes. Following habituation, a stimulus mouse of the same sex was placed in the enclosure with the test mouse. Interactions between the two mice were filmed and manually scored by an investigator masked to the experimental conditions using Stopwatch+™ (Centre for Behavioural Neuroscience, Georgia State University). The time spent sniffing the anogenital region of the other mouse, time sniffing the head-torso region, the time mice spent following one another and the time spent where both mice were sniffing in a reciprocal circle, was recorded.

#### Elevated plus maze

When mice were 43 days old, they performed a standard elevated plus maze (EPM) task to measure anxiety-like behaviour [102]. The arena was a ‘plus’ symbol shape with 30 cm long and 6 cm wide arms raised 39 cm from the ground. It contained two opposite “closed” arms that had 15 cm high walls and two opposite “open” arms, without walls. One at a time, mice were placed in the centre of the EPM arena, and their movement was tracked using TopScan Lite Software for five minutes. Time spent in the closed arms and time spent in the open arms was recorded, as well as the number of times the mouse entered each arm. Time in the centre region was excluded from analysis.

#### Hot/Cold plate

The hot/cold plate was used to assess thermal nociception and was performed on P44 [57]. The hot/cold apparatus was a 15 cm diameter enclosure with clear walls. Mice were habituated to the hot/cold apparatus for 5 min/day for two days prior to testing. On the morning of P44, mice were placed one at a time in the apparatus with the plate heated to 52^o^C, and the time it took for a hind paw withdrawal was scored. As soon as mice responded they were removed from the apparatus and returned to their home cage. At least one hour following testing on the hot plate, mice were again individually placed in the same apparatus, this time with the plate cooled to 2^o^C and the time to hind paw withdrawal was again recorded. Mice that did not respond within 2 min on the cold plate were removed. The apparatus was cleaned with Virkon between mice.

### Tissue collection and Preparation

Mice were humanely euthanised at an acute (P14), intermediate (P21), or chronic (P45) timepoint (Fig. 1C). On the day of euthanasia mice were deeply anaesthetised with 5% isoflurane in 2 L/min of oxygen. Mice were then injected i.p. with 0.16 mg/g of pentobarbitone.

#### Fresh tissue collection

Fresh brain tissue was extracted from half of the mice. The hippocampus (HPC) and prefrontal cortex (PFC) were isolated using the Paxinos and Franklin’s mouse brain atlas, collected, and snap frozen on dry ice for RNA extraction and qRT-PCR analysis. Tissue was homogenised using TissueLyser LT (Qiagen, Melbourne, Australia), a 5 mm stainless steel bead, and beta-mercaptoethanol, for five minutes at 50 Hz. RNA was extracted using a QIAcube (Qiagen, Germany), QIAcube RNeasy^®^ Mini extraction kits according to the manufacturer’s protocol. RNA was reverse transcribed into cDNA using qScript™ XLT cDNA SuperMix (Quantabio, USA) following manufacturer’s instructions. Next, cDNA was prepared for real time qRT-PCR using Taqman gene expression assays (Thermofisher Scientific, USA) and processed using the Fluidigm^®^ BioMark system at the Medical Genomic Facility (Monash Health Translational Precinct, Clayton, Australia). Data were analysed using the Fluidigm^®^ analysis software. The concentration of each gene was calculated using the 2^−ΔΔCT^ method and normalised to the average value of two or three housekeeping genes (*Ywhaz*,* Ppia*,* Hprt*), after confirmation that the housekeeping genes were unaltered by treatment. If a housekeeping gene was modified by the injury, it was not used for normalisation in that analysis. Data are expressed as fold change relative to respective sham mice. Genes analysed are listed in Supplementary Table 1.

#### Fixed tissue collection

Half of the mice underwent a transcardial perfusion with 0.9% sodium chloride followed by 4% paraformaldehyde (PFA). Brains were extracted and post-fixed in 4% PFA for 24 h at 4^o^C, then transferred into 30% sucrose for 72 h at 4^o^C. Brains were embedded in optimal cutting temperature (OCT) and snap frozen with iso-pentane and stored at -80^o^C until they were cryo-sectioned.

Mouse brains were coronally sliced into 20$\:\mu\:$m sections starting at Bregma − 2.30. Two serial sections were collected and then 6 were discarded, then another 2 sections were collected on the next slide, to systematically sample the entire brain. Collected sections were mounted onto Superfrost + slides. This method allowed a systematic sample of the entire brain, generating 100 sections/brain on 50 slides. Brains were systematically stained for glial fibrillary acidic protein (GFAP), oligodendrocyte transcription factor 2 (Olig2), ionized calcium adapter molecule 1 (Iba1), aquaporin 4 (AQP4), antigen kiel 67 (Ki67), and suppressor of cytokine signalling 1 (SOCS1). Prior to primary antibody application, slides were incubated in Dako antigen retrieval for 20 min at 90^o^C, then blocked with 10% Normal Donkey Serum (NDS). Primary antibodies were diluted with 5% NDS and 0.1% Triton-X-100 in phosphate buffered saline (PBS) and were incubated on slide overnight at 4^o^C. Slides were then washed with PBS and incubated at room temperature for one hour with the appropriate secondary antibody. Hoechst (1:1000) was used as a nuclear stain and slides were cover-slipped with Dako Aqueous Mounting Media. For a complete list of primary and secondary antibodies see Supplementary Table 2. Slides were stained in batches containing all experimental groups. Positive and negative control slides were also stained concurrently for each histological preparation.

### Image collection and analysis

#### Prefrontal cortex

All images were taken on a Leica Thunder microscope at 20x magnification and analysed using Fiji2 version 2.14.0 software. The PFC was imaged, and both the medial PFC (mPFC) and lateral PFC (lPFC) were analysed. The lPFC and mPFC were defined as in Supplementary Table 3, with the left and right lPFC averaged for each section. Sections containing the PFC were stained with GFAP, Iba1, and AQP4. Starting at approximately Bregma + 2.10, one slide every 420 $\:\mu\:$m was stained, resulting in 4 images of the mPFC and lPFC. For GFAP analysis, a threshold was set to 18, then the percent positive GFAP signal was automatically calculated within the regions of interest and averaged across the four sections. Images stained with AQP4 had a threshold set to 24, and total fluorescence and percent coverage of positive signal was calculated within the regions of interest and averaged across sections. All thresholds were determined relative to background signal using a series of blinded sample tissues including both sexes and all injury groups. Regarding Iba1, positively labelled cells were manually counted within the regions of interest.

#### Hippocampus

To analyse cells in the HPC, two consecutive sections were stained every 700$\:\:\mu\:$m starting at Bregma + 1.30, and only one of the two consecutive sections were imaged, resulting in three images acquired and analysed of the HPC with GFAP, Olig2, Iba1, AQP4, Ki67, and SOCS1. The HPC was manually traced on each image based on the Hoechst signal and defined as the region of interest. Images stained with GFAP had a threshold set to 14, and the percent coverage of GFAP positive signal within the region of interest was calculated. AQP4 and SOCS1 had thresholds set to 30 and 23, respectively, and the total fluorescence of each stain was calculated for each image. Fluorescence across the three images was averaged for each stain to give an average signal across the whole HPC. Regarding Olig2, Iba1, and Ki67, positively labelled cells of each individual stain were manually counted within the region of interest. Counts across the three images were summed to give ‘total’ cell counts for the HPC.

#### Corpus callosum

The corpus callosum (CC) was stained with Olig2 every 420$\:\:\mu\:$m starting at approximately Bregma + 2.10 and three images (one per section) were collected for each animal. Only the anterior CC when both hemispheres were connected were imaged and analysed. The CC was manually traced using Hoechst and total Olig2 positive cells were manually counted and summed for each brain.

#### Blood brain barrier integrity

Mice were examined for blood brain barrier (BBB) permeability using a modified method [100]. One hour before euthanasia, on P21, mice were anaesthetised with 4% isoflurane and 2.5 mg/mouse of tetramethylrhodamine (TMR) conjugated 70 kDa dextran (approximately the size of albumin; Invitrogen-Thermo Fisher Scientific, Waltham, MA) was injected into the retro-orbital socket of a single eye. Mice were allowed to recover for one hour before undergoing a transcardial perfusion; brains were then post-fixed and cryo-sectioned as described above. Sections were stained with Isolectin B4 (Ib4) conjugated to FITC used to indicate blood vessels. Images were analysed by setting a threshold to 14 for both Ib4 and TMR, and the fluorescence intensity of each label was calculated for the entire brain section. The fluorescence intensity for Ib4 was then subtracted from the fluorescence intensity of TMR to give the amount of TMR leakage from the vessels. This was repeated for the entire brain starting at approximately Bregma + 2.10 for 9 sections, with one image every 840$\:\:\mu\:$m to give a systematic sample of the entire brain.

### Statistical analysis

Statistics were analysed using two-way ANOVAs with injury (1, 3, 5, sham) and sex (male: female) as factors. When there were significant effects of injury, Least Significant Difference (LSD) post-hoc analyses were performed. All statistics were run on SPSS version 27 and significance was set at *p* < 0.05 unless otherwise stated. The full statistical output for gene expression is only reported when there were significant effects, but full raw data and statistical analyses are available at the open-source framework site https://osf.io/2kg5j/?view_only=945c457a3bdd4cb88294f04fdbd461c0 upon publication. Further, for the majority of immunohistochemical factors analysed, there were no sex effects. Therefore, statistical outputs from the two-way ANOVA for sex and the interaction effects of sex and injury are only presented here if significant, but full raw data and statistics are available at the open-source repository.

## Results

### 15s AHT (0, 1, and 3 Injuries)

#### Body and organ weights were unaffected by 15s injury.

**Fig. 2:**
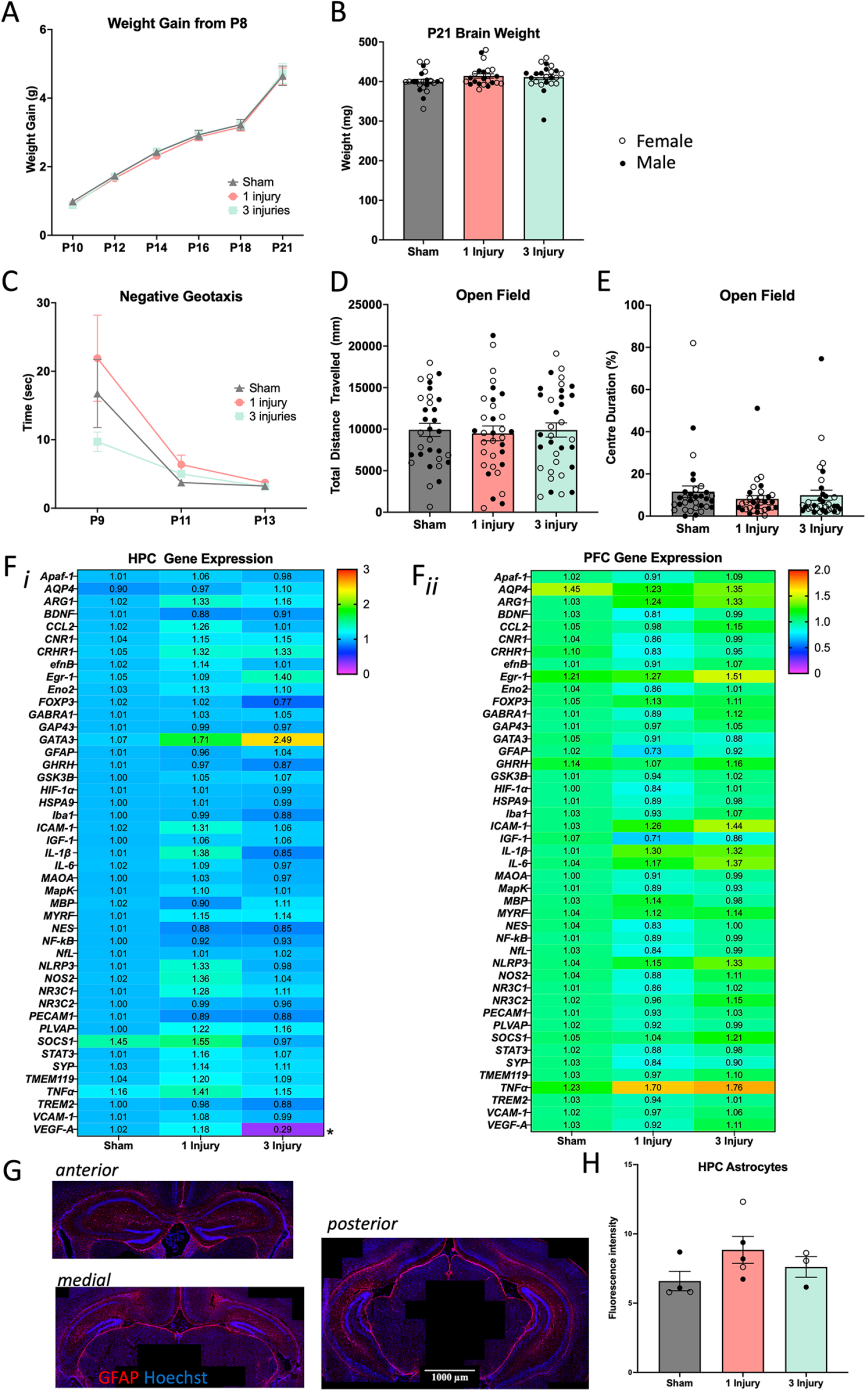
15s injury results. (**A**) Weight gain from P8 to euthanasia at P21. (**B**) Brain weight of female and male mice at euthanasia on P21. (**C**) Time for mice to turn upright on negative geotaxis on P9, P11 and P13. (**D**) Total distance travelled during the open field task and (**E**) the percent of time they spent within the centre zone of the open field. (**F**) Heat map of relative gene expression measured with PCR in the **(F*i*)** HPC and **(F*ii*)** PFC after a 15s injury. Genes are in alphabetical order and were normalised to 3 housekeeping genes. Asterisks indicate a significant main effect of injury on gene expression. (**G**) Representative examples of the anterior, medial, and posterior HPC stained with GFAP (red) and Hoechst (blue) taken from a mouse with a single injury. Images were taken at 20x magnification. (**H**) Percent coverage of positive GFAP signal in the whole HPC. Error bars are $\:\pm\:$SEM; females are denoted with open circles, and males are denoted with closed circles. Significance at *p* < 0.05 is denoted with *, and *p* < 0.01 with **

Mice were weighed every alternate day from P8 until sacrifice at P21. A two-way repeated measures ANOVA with Mauchly’s test of sphericity was used to examine differences in body weight between sex and injury group over time. There was a significant increase in body weight over time (F(1,93) = 1492.90, *p* < 0.001), as expected; however, there were no significant differences in weights across injury or sex at any of the time points measured (Fig. 2A). Whole fresh brains and spleens were weighed following euthanasia on P21. Using a two-way ANOVA, brain weight did not differ by sex or injury group (Fig. 2B). Spleens weighed significantly less in females compared to males, but there was no effect of injury or an interaction effect on spleen weight (Table 1). Full statistical analyses for weight and behaviours are listed in Table 1.

### No behavioural deficits were produced by the 15s injury

#### Negative geotaxis and open field

Mice were tested for proprioception using the negative geotaxis task on P9, P11, and P13. For each of the 3 test days, there were no effects of sex or injury, nor was there a significant interaction (Fig. 2C; Table 1). On P18, mice were also tested for general locomotion and anxiety-like behaviour using the open field task. There were no significant effects of sex or injury in the total distance travelled (Fig. 2D) or velocity. The percentage of time the mice spent in the centre zone (Fig. 2E) was not affected by sex or by injury, nor was the time the mice spent in the outer zone. The number of times the mouse entered the centre of the arena was also not significantly altered by sex or injury (Table 1).

**Table 1:** Statistical results from the two-way ANOVA for weights and behavioural tasks

Measure		ANOVA Results		
		Sex	Injury	Interaction
Weight gain from P8	P10	F(1,119) = 0.02 *p* = 0.89	F(2,119) = 0.51 *p* = 0.61	F(2,119) = 1.06 *p* = 0.35
	P12	F(1,119) = 0.28 *p* = 0.60	F(2,119) = 0.28 *p* = 0.76	F(2,119) = 0.51 *p* = 0.60
	P14	F(1,119) = 0.10 *p* = 0.76	F(2,119) = 0.19 *p* = 0.83	F(2,119) = 1.41 *p* = 0.25
	P16	F(1,119) = 0.02 *p* = 0.89	F(2,119) = 0.76 *p* = 0.47	F(2,119) = 1.13 *p* = 0.33
	P18	F(1,119) = 0.09 *p* = 0.79	F(2,119) = 0.79 *p* = 0.46	F(2,119) = 1.11 *p* = 0.33
	P21	F(1,119) = 0.05 *p* = 0.82	F(2,119) = 1.47 *p* = 0.87	F(2,119) = 0.99 *p* = 0.38
P21 brain weight		F(1,78) = 0.67 *p* = 0.42	F(2,78) = 1.46 *p* = 0.24	F(2,78) = 0.14 *p* = 0.87
P21 spleen weight		F(1,78) = 4.08 *p* = 0.047 ♂>♀	F(2,78) = 0.09 *p* = 0.91	F(2,78) = 2.31 *p* = 0.11
Negative geotaxis	P9	F(1,98) = 0.29 *p* = 0.59	F(2,98) = 2.17 *p* = 0.12	F(2,98) = 0.06 *p* = 0.94
	P11	F(1,98) = 2.50 *p* = 0.12	F(2,98) = 0.04 *p* = 0.96	F(2,98) = 0.01 *p* = 0.91
	P13	F(1,98) = 2.91 *p* = 0.14	F(2,98) = 2.24 *p* = 0.11	F(2,98) = 0.09 *p* = 0.92
Open field	Total distance	F(1,98) = 0.34 *p* = 0.56	F(2,98) = 0.08 *p* = 0.93	F(2,98) = 0.18 *p* = 0.83
	Velocity	F(1,98) = 1.63 *p* = 0.21	F(2,98) = 1.01 *p* = 0.37	F(2,98) = 0.91 *p* = 0.41
	Centre bouts	F(1,98) = 0.51 *p* = 0.48	F(2,98) = 0.10 *p* = 0.91	F(2,98) = 0.01 *p* = 0.98
	Centre %	F(1,98) = 0.001 *p* = 0.97	F(2,98) = 0.52 *p* = 0.59	F(2,98) = 0.23 *p* = 0.80

### Brain gene expression and immunohistochemistry largely unaffected from injury

#### HPC gene expression

On P21 the HPC was isolated, and RNA was extracted from sham injured mice, 1 injury mice, and 3 injury mice. High throughput qRT-PCR on 45 genes of interest was used to examine differences in gene expression in female (*n* = 13) and male (*n* = 10) mice (Fig. 2F*i*). There was a significant reduction of *VEGF-A* expression due to injury; however, there were no other significant alterations in gene expression. Statistical analysis of genes that were significantly altered are displayed in Table 2.

#### PFC gene expression

Relative gene expression of the same genes analysed in the HPC were also examined in the PFC of P21 mice (Fig. 2F*ii*). Here, there were sex by injury interaction on the expression of *CNR1*,* CRHR1*,* Eno2*,* MapK*, and *SYP* whereby in general they were significantly reduced in injured mice, and this effect was likely driven by a reduction in expression in female mice (Table 2). *GABRA1* was significantly increased from 3 injuries compared to a single injury, *HIF-1*$\:\alpha\:$ was significantly reduced due to injury, and *GAP43* and *MBP* both exhibited reduced expression in females compared to males. There was high variance in some gene expression data (e.g., GATA3), and there was no other significant effects on gene expression.

**Table 2:** P21 PCR results following 15s injuries. Statistical results from the two-way ANOVA for gene expression in the HPC and PFC following 1, 3 or sham 15s injuries

Gene	ANOVA Results		
	Sex	Injury	Interaction
HPC			
*VEGF-A*	F(1,22) = 0.04 *p* = 0.84	F(2,22) = 10.56 *p* = 0.001 0 > 3, *p* = 0.004 1 > 3, *p* < 0.001	F(2,22) = 0.29 *p* = 0.75
PFC			
*CNR1*	F(1,22) = 0.001 *p* = 0.98	F(2,22) = 1.41 *p* = 0.27	F(2,22) = 6.31 *p* = 0.01 ♀0>♂0, *p* = 0.03 ♀0>♀1, *p* = 0.04 ♀0>♀3, *p* = 0.01
*CRHR1*	F(1,22) = 1.93 *p* = 0.18	F(2,22) = 3.25 *p* = 0.06	F(2,22) = 6.21 *p* = 0.01 ♀0>♂0, *p* = 0.01 ♀0>♀1, *p* = 0.03 ♀0>♀3, *p* = 0.03
*Eno2*	F(1,22) = 0.35 *p* = 0.56	F(2,22) = 1.97 *p* = 0.27	F(2,22) = 9.15 *p* = 0.002 ♀0>♂0, *p* = 0.02 ♀0>♀1, *p* = 0.01 ♀0>♀3, *p* = 0.01 ♂0<♂3, *p* = 0.02 ♂1<♂3, *p* = 0.01
*GABRA1*	F(1,22) = 1.05 *p* = 0.32	F(2,22) = 3.90 *p* = 0.04	F(2,22) = 0.17 *p* = 0.84
*GAP43*	F(1,22) = 5.26 *p* = 0.04 ♀<♂	F(2,22) = 0.32 *p* = 0.73	F(2,22) = 0.71 *p* = 0.51
*HIF-1* $\:\alpha\:$	F(1,22) = 0.32 *p* = 0.58	F(2,22) = 3.71 *p* = 0.05	F(2,22) = 0.17 *p* = 0.84
*MapK*	F(1,22) = 0.66 *p* = 0.43	F(2,22) = 2.26 *p* = 0.14	F(2,22) = 4.97 *p* = 0.02
*MBP*	F(1,22) = 5.60 *p* = 0.03 ♀<♂	F(2,22) = 0.48 *p* = 0.63	F(2,22) = 0.44 *p* = 0.65
*SYP*	F(1,22) = 0.04 *p* = 0.85	F(2,22) = 4.58 *p* = 0.03	F(2, 22) = 14.13 *p* < 0.001 ♀0>♂0, *p* = 0.003 ♀3<♂3, *p* < 0.001

#### HPC astrocyte immunohistochemistry

Sections of the HPC (Fig. 2G) were stained for GFAP and evaluated for percentage positive signal coverage at P21. The three regions were averaged to give a measure of the whole HPC, where there were no significant main effects of sex (F(1,11) = 0.99, *p* = 0.36), injury (F(2,11) = 2.78, *p* = 0.14), nor a significant interaction (F(2,11) = 1.89, *p* = 0.23), in GFAP signal (Fig. 2H).

### 60s AHT (Sham, 1, 3, and 5 Injuries)

#### Brain but not body weight was altered by AHT

**Fig. 3:**
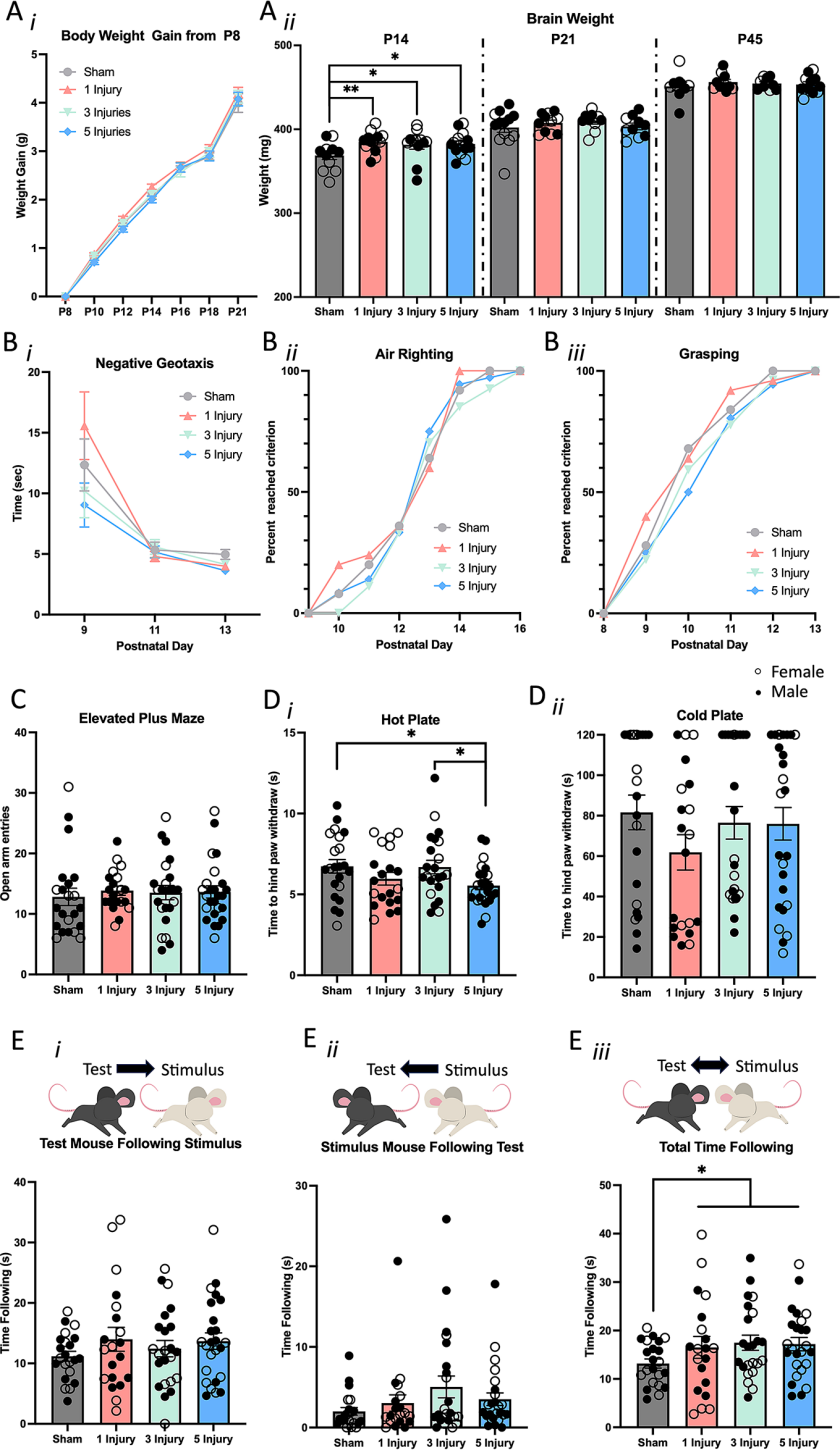
Weight, acute behaviour, and chronic behaviour following 0, 1, 3, or 5 60s injuries. (**A*i***) Body weight gain of mice from P8 until P21. (**A*ii*)** Fresh brain weight of mice at P14, P21, and P45. (**B**) Acute behavioural results for (**B*i*)** time for mice to turn upright on negative geotaxis on P9, P11 and P13 (**B*ii***) mice to successfully land on all 4 paws for 2 consecutive days, and (**B*iii***) mice to hang by their forelimbs for more than 1s for 2 consecutive days. (**C**) The amount of open arm entries of mice on the elevated plus maze. (**D**) Time for mice to react with a hind paw withdrawal on the (**D*i***) hot plate and (**D*ii***) cold plate. (**E**) Total time in the social interaction that (**E*i***) test mice followed the stimulus mice (**E*ii***) stimulus mice followed the test mice, and (**E*iii***) both mice spent following one another. Error bars are $\:\pm\:$SEM; females are denoted with open circles, and males are denoted with closed circles. Significance at *p* < 0.05 is denoted with *, and *p* < 0.01 denoted with **

Mice were weighed from the start of injury on P8 until euthanasia. A two-way repeated measures ANOVA with Mauchly’s test of sphericity was used to examine differences in body weight over time (Fig. 3Ai). Weight significantly increased over time (F(6,195) = 1984.92, *p* < 0.001), and males gained more weight than females (F(6, 195) = 2.78, *p* = 0.01); however, injury did not significantly alter weight gain (F(6, 195) = 0.78, *p* = 0.59). Whole fresh brains and spleens were weighed following euthanasia on P14, P21, and P45. The two-way ANOVA indicated that brains of female mice weighed significantly less at P14 compared to brains of male mice (F(1, 50) = 12.74, *p* < 0.001). Injury significantly altered P14 brain weight (F(1, 50) = 10.56, *p* = 0.02), with brains of mice with a single injury (*p* = 0.01), 3 injuries (p *=* 0.03), and 5 injuries (*p* = 0.02) weighing more than sham mice. However, the effect of injury on brain weight was not evident at P21 (F(1, 43) = 1.20, *p* = 0.28) or P45 (F(1, 40) = 0.34, *p* = 0.56). Compared to the female brain, the male brain weighed more at P21 and less at P45 (F(1, 43) = 15.16, *p* < 0.001, F(1, 40) = 4.18, *p* = 0.048, respectively). Spleen weight did not change as a result of sex or injury at P14 (F(1, 85) = 0.68, *p* = 0.41, F(1, 85) = 0.07, *p* = 0.79, respectively), nor at P21 (F(1, 82) = 0.003, *p* = 0.96), F(1, 82) = 0.09, *p* = 0.77, respectively).

### Acute behaviour was unaffected by AHT

Acute behavioural deficits were examined using negative geotaxis, air righting, and grasping. Results were analysed using a two-way ANOVA to compare sex and injury; however, sex did not alter acute behaviours analysed, thus the statistical output for these are not displayed here, and are available at the opensource framework stated above. The time for the mice to turn to face up on negative geotaxis was not altered by injury on any of the three days tested (F(1, 173) = 0.05, *p* = 0.83; F(1, 173) = 0.02, *p* = 0.88; F(1,173) = 2.17, *p* = 0.14; for P9, P11, and P13, respectively; Fig. 3Bi). Furthermore, there was no effect of injury on air righting (F(1, 112) = 0.08, *p* = 0.78; Fig. 3Bii), nor on grasping (F(1, 112) = 0.69, *p* = 0.41; Fig. 3Biii).

**Fig. 4:**
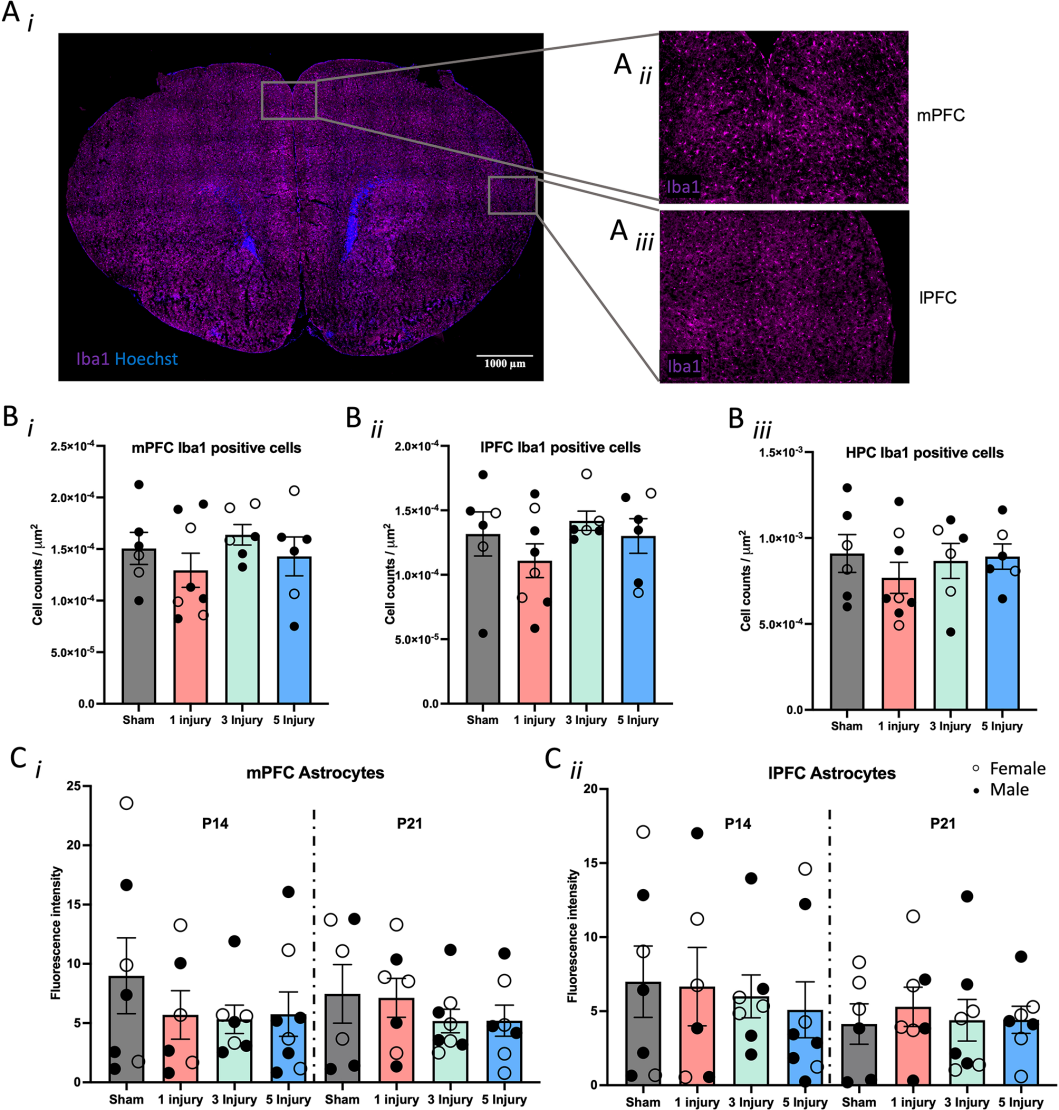
Immunohistochemistry results from Iba1 and GFAP following 0, 1, 3, or 5, 60s injuries. (**A**) Representative example of a brain stained with Iba1 (purple) and Hoechst (blue) of the **(A*i*)** whole slice, and sections blown up to show the **(Ai)** mPFC and **(Aii)** lPFC. Images were taken at 20x magnification and scale bar indicates 1000 $\:\mu\:$m. (**B**) Counts of Iba1 positively stained cells in the (**B*i***) mPFC, (**B*ii***) lPFC, and (**B*iii***) HPC. (**C**) Percent coverage of GFAP positive astrocytes in the (**C*i***) mPFC and (**C*ii***) lPFC in P14 and P21 mice. Error bars are ± SEM; females are denoted with open circles, and males are denoted with closed circles. Significance at *p* < 0.05 is denoted with *, and *p* < 0.01 with **

### Chronic behaviour

#### Anxiety-like behaviours were unaffected by AHT

Adolescent mice were tested for behavioural deficits on the open field task, social interaction task, hot/cold plate, and EPM. Open field was performed on P18 and P45 to measure general locomotion and anxiety-like behaviours. There was no significant main effect of injury on the total distance mice travelled at either time point (P18: F(1, 106) = 1.54, *p* = 0.22, P45: F(1, 87) = 3.55, *p* = 0.06). However, on P18, females travelled further than males (F(1, 106) = 6.62, *p* = 0.01), while the opposite occurred at P45, with males travelling increased distances (F(1, 87) = 7.03, *p* = 0.01; data not displayed here). Regarding anxiety-like behaviours in the open field, there was no effect of injury on the amount of time mice spent in the centre at P18 or P45 (F(1, 106) = 0.98, *p* = 0.32, F(1, 87) = 0.39, *p* = 0.54, respectively), or the distance travelled within the centre zone (F(1, 106) = 1.01, *p* = 0.32, F(1, 87) = 1.57, *p* = 0.21, respectively). The EPM also indicated no difference in anxiety like behaviours from injury, with both the number of open arm entries (Fig. 3C) and time spent in the open arms being non-significant (F(1, 89) = 0.50, *p* = 0.48, F(1, 89) = 0.70, *p* = 0.41, respectively).

#### Nociceptive thresholds were reduced in AHT mice

Thermal nociception was examined in adolescent mice using the hot/cold plate at P44. On the hot plate, injury increased heat sensitivity compared to sham mice (F(1, 89) = 2.76, *p* = 0.048) such that the mice with 5 injuries exhibited decreased reaction time compared to sham mice (*p* = 0.02) and compared to mice with 3 injuries (*p* = 0.03; Fig. 3Di). Conversely, sensitivity to cold stimuli remained unaffected by injury (F(1, 89) = 1.04, *p* = 0.31, Fig. 3Dii).

#### Social behaviours largely unaffected by AHT

Social behaviours were assessed by introducing experimental mice to a sex-matched naïve mouse and quantifying interactions. Time mice spent sniffing the head and torso region of the stimulus mouse was recorded, and was not affected by injury (F(1, 89) = 0.02, *p* = 0.90), nor was the time mice spent sniffing the anogenital region of the stimulus mouse (F(1, 89) = 0.33, *p* = 0.57). Time mice spent following each other was recorded and the duration the test mouse followed the stimulus mouse (Fig. 3Ei). The duration the stimulus mouse followed the test mouse (Fig. 3Eii) was not significantly altered by injury (F(1, 89) = 1.70, *p* = 0.20, F(1, 89) = 2.82, *p* = 0.10, respectively). The total time mice spent following one another (Fig. 3Eiii) had a main effect of injury (F(1, 89) = 5.11, *p* = 0.03). Subsequent post-hoc tests indicated this effect was driven by sham mice having a trend for reduced following time compared to mice in the single injury group (*p* = 0.09), 3 injury group (*p* = 0.07), and 5 injury group (*p* = 0.06).

### Immunohistochemistry

#### Glial reactivity (Iba1 and GFAP) was unaltered from AHT at P14 and P21 in the PFC and HPC

Brains from P14 and P21 mice were cryo-sectioned and stained for GFAP and Iba1 as markers of neuroinflammation in the mPFC, lPFC, and HPC. Regarding Iba1 (Fig. 4A*i*-*iii*), a two-way ANOVA examining sex and injury factors in the P21 brain revealed that injury did not alter the number of Iba1 positive cells in the mPFC (F(1, 27) = 0.26, *p* = 0.61; Fig. 4B*i*), lPFC (F(1, 27) = 2.44, *p* = 0.13; Fig. 4B*ii*), or in the HPC (F(1, 27) = 0.63, *p* = 0.44; Fig. 4B*iii*). The same regions were examined for astrocyte expression using GFAP at P14 and P21. Injury did not alter astrocytes in the mPFC at P14 (F(1, 27) = 2.08, *p* = 0.17), or at P21 (F(1, 27) = 0.59, *p* = 0.45; Fig. 4C*i*). Similar to the mPFC, astrocyte expression did not change from injury in the lPFC at P14 or P21 (F(1, 27) = 0.22, *p* = 0.65, F(1, 27) = 0.26, *p* = 0.61, respectively; Fig. 4C*ii*). Finally, GFAP was not altered by injury at P14 or P21 in the HPC (F(1, 27) = 0.15, *p* = 0.70, F(1, 27) = 0.15, *p* = 0.70, respectively).

#### Cell birth and death (SOCS1 and Ki67) in the HPC was not affected by AHT at P14 or P21

**Fig. 5:**
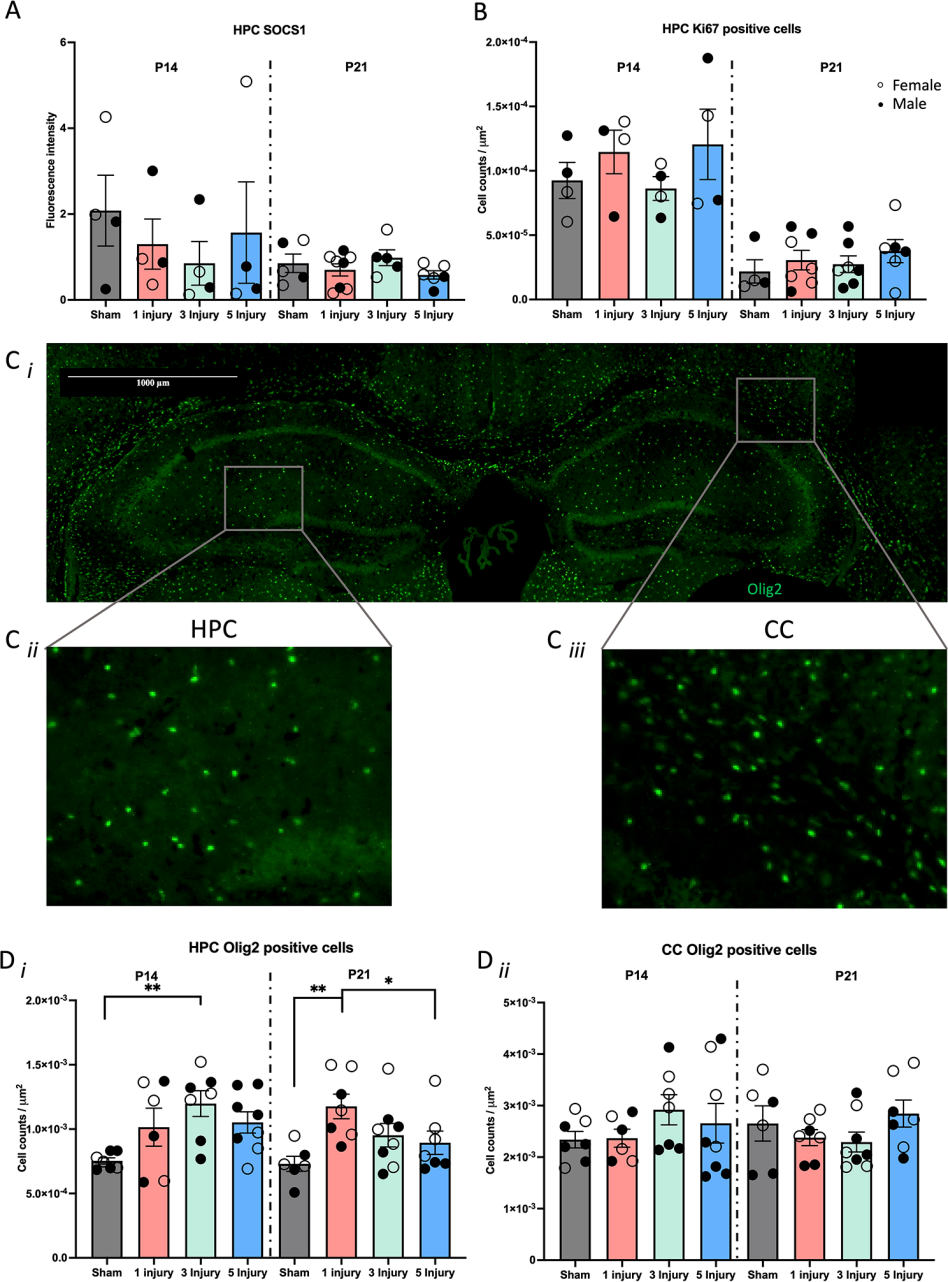
Immunohistochemistry results from SOCS1, Ki67, and Olig2 following 0, 1, 3, or 5, 60s injuries. (**A**) Cell death in the P14 and P21 HPC quantified with percent converge of SOCS1 positive signal. (**B**) Cell birth in the P14 and P21 HPC signified by counting Ki67 positive cells. (**C*i***) Representative image of Olig2 staining (green) in the (**C*ii***) HPC, and **(C*iii***) section of the CC. (**D**) Olig2 positive cell counts from the P14 and P21 (**D*i***) HPC and (**D*ii***) CC. Error bars are ± SEM; females are denoted with open circles, and males are denoted with closed circles. Significance at *p* < 0.05 is denoted with *, and *p* < 0.01 with **

Cell birth and death was quantified within the P14 and P21 HPC. SOCS1 inhibits cell proliferation and promotes apoptosis [85]. Regarding cell death, SOCS1 signal fluorescence was calculated in the HPC of P14 and P21 mice. SOCS1 fluorescence was not affected by injury at P14 (F(1, 15) = 0.88, *p* = 0.37), or at P21 (F(1, 23) = 0.83, *p* = 0.37); Fig. 5A). Ki67 was used to stain newly divided cells, and positively stained cells were manually counted. There was no difference in cell birth from injury at P14 (F(1, 15) = 0.48, *p* = 0.50), or at P21(F(1, 23) = 1.28, *p* = 0.27; Fig. 5B).

#### White matter (Olig2) was increased in the P14 and P21 HPC but not the CC due to AHT

Oligodendrocytes were stained with an Olig2 antibody and manually counted within the HPC and CC (Fig. 5C*i*–*iii*). Injuries increased the number of Olig2 positive cells within the HPC at P14 (F(1,27) = 8.67, *p* = 0.01) and at P21 (F(1,27) = 5.24, *p* = 0.03; Fig. 5D*i*). This effect was driven by mice with 3 injuries having increased Olig2 cells compared to sham mice (*p* = 0.001) and a trend for increased cells in the single injury (*p* = 0.06) and 5 injury mice (*p* = 0.05) compared to sham mice. At P21, the injury effect was indicated by single injury mice having increased Olig2 positive cells compared to sham mice (*p* = 0.004) and 5 injury mice (*p* = 0.04). Conversely, in the CC, Olig2 positive cells were not altered by injury at P14 (F(1,27) = 0.60, *p* = 0.45), nor at P21 (F(1,27) = 0.27, *p* = 0.61; Fig. 5D*ii*).

#### BBB permeability (AQP4, Ib4, and TMR) was increased from AHT

**Fig. 6:**
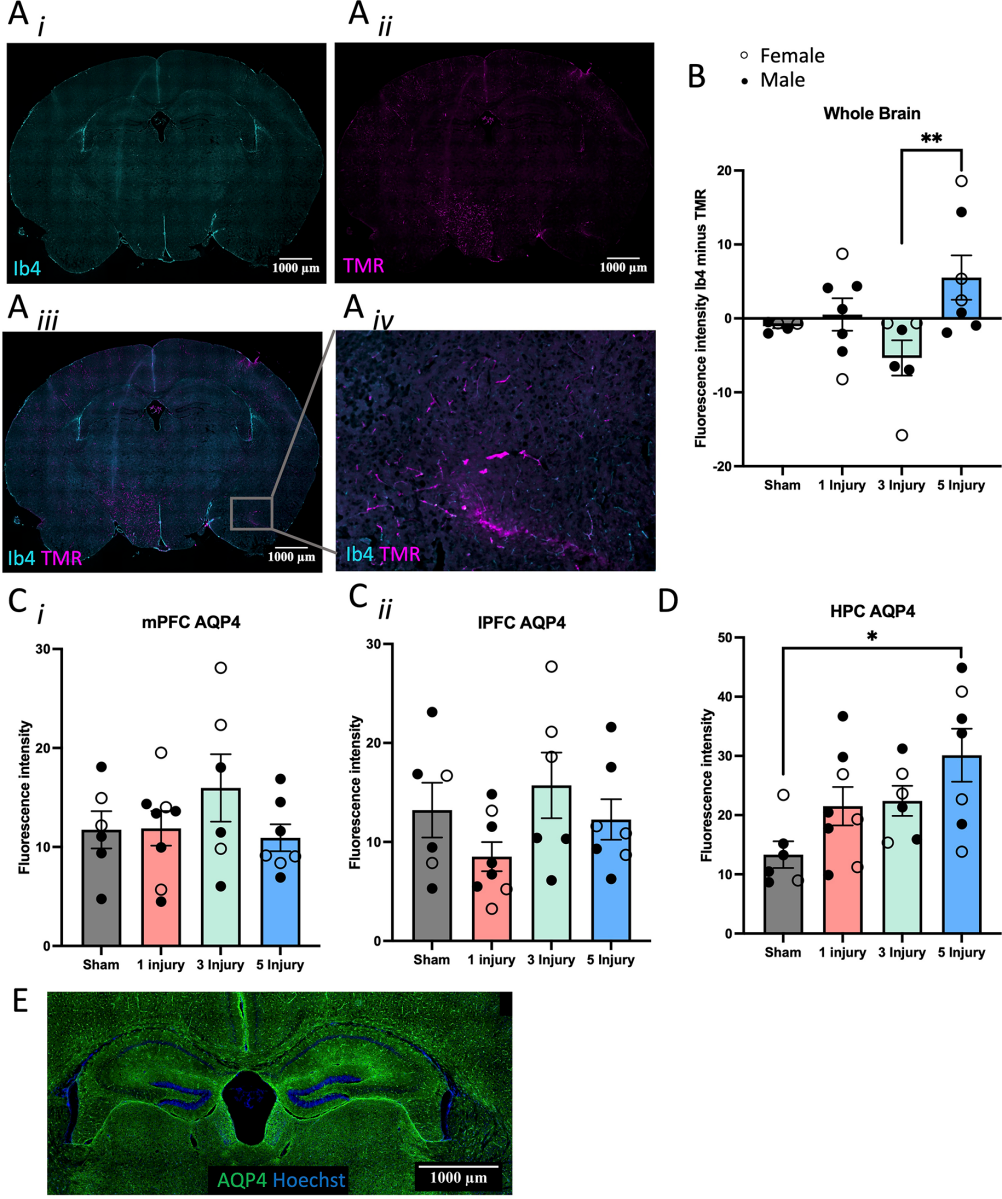
BBB permeability measurements in P21 mice following 0, 1, 3 or 5 60s injuries. (**A**) Representative example of whole brain slices stained with (**A*i***) Ib4 (cyan) to label blood vessels, (**A*ii***) fluorescent dye, TMR (magenta), (**A*iii***) both Ib4 and TMR, and (**A*iv***) close up of Ib4 and TMR to show TMR leakage from the vessels. Images were taken at 20x magnification. (**B**) Quantification of TMR leakage from vessels by subtracting the fluorescence of Ib4 from the fluorescence of TMR. (**C**) Quantification of AQP4 fluorescence in the (**C*i***) mPFC, (**C*ii***) lPFC, and (**D**) HPC of female and male mice. (**E**) Representative example of AQP4 (green) staining and Hoechst (blue) in the HPC. Images were taken at 20x magnification and scale bars indicate 1000 $\:\mu\:$m. Error bars are ± SEM; females are denoted with open circles, and males are denoted with closed circles. Significance at *p* < 0.05 is denoted with *, and *p* < 0.01 with **

BBB integrity was examined using a blood vessel label (Ib4) and TMR (Fig. 6A*i*-*iv*). Injuries significantly altered vascular leakage of TMR (F(1, 24) = 3.39, *p* = 0.04), with 5 injuries resulting in increased leakage compared to 3 injuries (*p* = 0.01), and a trend of increased leakage compared to the sham injured mice (*p* = 0.09; Fig. 6B). To further explore this, the fluorescence intensity of a water channel protein linked to brain oedema in models of TBI, AQP4, was examined in the mPFC, lPFC, and HPC in P21 mice. AQP4 expression was not altered in the mPFC (F(1, 26) = 0.63, *p* = 0.81; Fig. 6C*i*). In the lPFC neither sex (F(1, 26) = 0.62, *p* = 0.44), nor injury (F(1, 26) = 2.22, *p* = 0.12) independently altered AQP4 expression; however, there was a sex by injury interaction (F(1, 26) = 3.35, *p* = 0.04), such that female mice with 3 injuries had significantly increased AQP4 expression compared to male mice with 3 injuries (*p* = 0.01). In female mice there was increased AQP4 expression in mice with 3 injuries compared to single injury mice and 5 injury mice (*p* = 0.003, *p* = 0.01, respectively), and a trend for increased expression compared to sham mice (*p* = 0.05; Fig. 6C*ii*). In the HPC, there was a significant effect of injury (F(1, 26) = 4.51, *p* = 0.045; Fig. 6D), whereby mice in the 5 injury group had increased AQP4 expression compared to sham injured mice (*p* = 0.01; Fig. 6E).

### Gene expression

#### P14 gene expression was altered in a region dependent manner

**Fig. 7:**
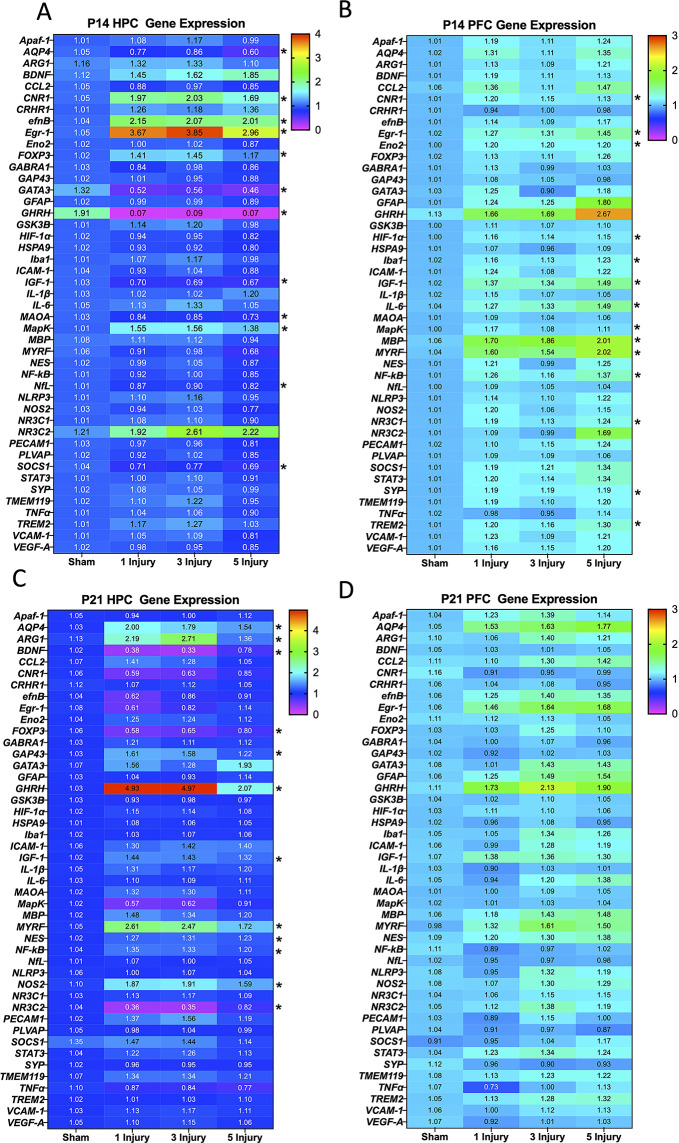
60s PCR results from the P14 and P21 HPC and PFC. (**A**) Heatmap of genes examined in the P14 HPC and (**B**) PFC. (**C**) Heatmap of relative gene expression in the P21 HPC and (**D**) PFC. Significant changes (*p* < 0.01) in gene expression due to injury are indicated with *. Results are displayed as means of combined female and male mice and genes are listed in alphabetical order

RNA was extracted and isolated from the HPC and PFC as previously described, and high throughput qRT-PCR was used to examine expression of 45 genes between injury groups and sex. A two-way ANOVA with sex and injury as factors was used. To account for multiple comparisons, significance was set as *p* < 0.01. There was a significant alteration in gene expression due to injury in 12/45 genes examined in the HPC and 14/45 genes in the PFC (Fig. 7A and B). Of note, all genes that had altered expression in the PFC were upregulated due to injury. In the HPC genes involved in growth (*GHRH*, and *IGF-1*) had decreased expression, while genes involved in nociceptive processing (*Egr-1*, and *MapK*) had increased expression following injury. Full statistical analysis of genes that were significantly affected by sex or injury are displayed in Table 3.

**Table 3:** P14 PCR results following 60s injuries. Statistical results from the two-way ANOVA for gene expression in the P14 HPC and PFC following 1, 3, 5, or Sham 60s injuries

Gene	ANOVA Results		
	Sex	Injury	Interaction
HPC			
*AQP4*	F(1,46) = 1.80 *p* = 0.19	F(1,46) = 6.85 *p* = 0.01 0 > 5, *p* = 0.003	F(1,46) = 0.01 *p* = 0.93
*CNR1*	F(1,46) = 0.38 *p* = 0.54	F(1,46) = 17.96 *p* < 0.001 0 < 1, *p* < 0.001 0 < 3, *p* < 0.001 0 < 5, *p* = 0.01	F(1,46) = 0.04 *p* = 0.83
*EfnB*	F(1,46) = 0.64 *p* = 0.64	F(1,46) = 12.75 *p* < 0.001 0 < 1, *p* = 0.003 0 < 3, *p* = 0.01 0 < 5, *p* = 0.01	F(1,46) = 1.50 *p* = 0.23
*Egr-1*	F(1,46) = 0.01 *p* = 0.92	F(1,46) = 17.28 *p* < 0.001 0 < 1, *p* < 0.001 0 < 3, *p* < 0.001 0 < 5, *p* = 0.01	F(1,46) = 0.01 *p* = 0.91
*FOXP3*	F(1,46) = 0.27 *p* = 0.61	F(1,46) = 8.49 *p* = 0.01 0 < 1, *p* = 0.002 0 < 3, *p* = 0.002	F(1,46) = 0.62 *p* = 0.44
*GABRA1*	F(1,46) = 5.31 *p* = 0.03 ♀>♂	F(1,46) = 5.28 *p* = 0.03	F(1,46) = 1.18 *p* = 0.09
*GATA3*	F(1,46) = 1.59 *p* = 0.22	F(1,46) = 20.02 *p* < 0.001 0 > 1, *p* = 0.001 0 > 3, *p* = 0.002 0 > 5, < 0.001	F(1,46) = 0.02 *p* = 0.88
*GFAP*	F(1,46) = 6.45 *p* = 0.02 ♀>♂	F(1,46) = 1.55 *p* = 0.22	F(1,46) = 0.62 *p* = 0.43
*GHRH*	F(1,46) = 0.82 *p* = 0.37	F(1,46) = 36.69 *p* < 0.001 0 > 1, *p* < 0.001 0 > 3, *p* < 0.001 0 > 5, *p* < 0.001	F(1,46) = 0.79 *p* = 0.38
*HSPA9*	F(1,46) = 2.63 *p* = 0.11	F(1,46) = 4.20 *p* = 0.05	F(1,46) = 0.23 *p* = 0.64
*IGF-1*	F(1,46) = 0.31 *p* = 0.58	F(1,46) = 4.20 *p* = 0.01 0 > 1, *p* = 0.01 0 > 3, *p* = 0.01 0 > 5, *p* = 0.004	F(1,46) = 0.01 *p* = 0.99
*MAOA*	F(1,46) = 2.16 *p* = 0.15	F(1,46) = 7.49 *p* = 0.01 0 > 5, *p* = 0.01	F(1,46) = 1.18 *p* = 0.28
*MapK*	F(1,46) = 0.01 *p* = 0.92	F(1,46) = 16.76 *p* < 0.001 0 < 1, *p* < 0.001 0 < 3, *p* < 0.001 0 < 5, *p* = 0.01	F(1,46) = 0.01 *p* = 0.95
*MBP*	F(1,46) = 4.47 *p* = 0.04 ♀>♂	F(1,46) = 0.18 *p* = 0.67	F(1,46) = 0.60 *p* = 0.45
*NfL*	F(1,46) = 5.44 *p* = 0.03 ♀>♂	F(1,46) = 7.42 *p* = 0.01 0 > 1, *p* = 0.05 0 > 5, *p* = 0.01	F(1,46) = 0.33 *p* = 0.57
*SOCS1*	F(1,46) = 0.01 *p* = 0.91	F(1,46) = 7.68 *p* = 0.01 0 > 1, *p* = 0.03 0 > 5, *p* = 0.01	F(1,46) = 0.01 *p* = 0.93
*SYP*	F(1,46) = 4.80 *p* = 0.03 ♀>♂	F(1,46) = 0.02 *p* = 0.89	F(1,46) = 0.25 *p* = 0.62
PFC			
*Apaf-1*	F(1,46) = 0.30 *p* = 0.98	F(1,46) = 4.41 *p* = 0.04	F(1,46) = 0.52 *p* = 0.48
*AQP4*	F(1,46) = 0.02 *p* = 0.90	F(1,46) = 5.09 *p* = 0.03	F(1,46) = 0.02 *p* = 0.89
*CCL2*	F(1,46) = 0.002 *p* = 0.97	F(1,46) = 3.89 *p* = 0.03	F(1,46) = 0.60 *p* = 0.62
*CNR1*	F(1,46) = 0.38 *p* = 0.55	F(1,46) = 4.25 *p* = 0.004 0 < 1, *p* = 0.002 0 < 3, *p* = 0.01 0 < 5, *p* = 0.03	F(1,46) = 0.91 *p* = 0.35
*Egr-1*	F(1,46) = 7.33 *p* = 0.01 ♀>♂	F(1,46) = 10.55 *p* = 0.002 0 < 1, *p* = 0.04 0 < 3, *p* = 0.04 0 < 5, *p* < 0.001	F(1,46) = 1.88 *p* = 0.18
*Eno2*	F(1,46) = 1.98 *p* = 0.17	F(1,46) = 21.38 *p* < 0.001 0 < 1, *p* < 0.001 0 < 3, *p* < 0.001 0 < 5, *p* < 0.001	F(1,46) = 1.04 *p* = 0.31
*GFAP*	F(1,46) = 0.20 *p* = 0.66	F(1,46) = 4.83 *p* = 0.03	F(1,46) = 0.06 *p* = 0.81
*GHRH*	F(1,46) = 2.71 *p* = 0.11	F(1,46) = 5.11 *p* = 0.03	F(1,46) = 3.96 *p* = 0.02
*GSK3B*	F(1,46) = 0.08 *p* = 0.78	F(1,46) = 3.10 *p* = 0.04	F(1,46) = 1.23 *p* = 0.31
*HIF-1* $\:\alpha\:$	F(1,46) = 1.34 *p* = 0.25	F(1,46) = 6.60 *p* = 0.001 0 < 1, *p <* 0.001 0 < 3, *p* = 0.002 0 < 5, *p <* 0.001	F(1,46) = 0.34 *p* = 0.80
*Iba1*	F(1,46) = 0.06 *p* = 0.81	F(1,46) = 6.74 *p* = 0.01 0 < 5, *p* = 0.01	F(1,46) = 0.94 *p* = 0.34
*IGF-1*	F(1,46) = 0.80 *p* = 0.38	F(1,46) = 5.19 *p* = 0.03	F(1,46) = 0.02 *p* = 0.89
*IL-6*	F(1,46) = 0.50 *p* = 0.50	F(1,46) = 8.18 *p* = 0.01 0 < 3, *p* = 0.04 0 < 5, *p* = 0.003	F(1,46) = 2.29 *p* = 0.14
*MapK*	F(1,46) = 0.01 *p* = 0.94	F(1,46) = 5.08 *p* = 0.01 0 < 1, *p* < 0.001 0 < 5, *p* = 0.01	F(1,46) = 1.87 *p* = 0.15
*MBP*	F(1,46) = 0.84 *p* = 0.36	F(1,46) = 14.76 *p* < 0.001 0 < 1, *p* = 0.02 0 < 3, *p* = 0.01 0 < 5, *p* < 0.001	F(1,46) = 0.49 *p* = 0.49
*MYRF*	F(1,46) = 1.86 *p* = 0.18	F(1,46) = 9.02 *p* = 0.004 0 < 1, *p* = 0.05 0 < 5, *p* < 0.001	F(1,46) = 0.02 *p* = 0.90
*NES*	F(1,46) = 0.26 *p* = 0.61	F(1,46) = 4.57 *p* = 0.04	F(1,46) = 0.31 *p* = 0.58
*NF-kB*	F(1,46) = 0.63 *p* = 0.43	F(1,46) = 4.26 *p* = 0.01 0 < 1, *p* = 0.03 0 < 5, *p* = 0.001 3 < 5, *p* = 0.04	F(1,46) = 0.95 *p* = 0.43
*NLRP3*	F(1,46) = 0.4.64 *p* = 0.04 ♀<♂	F(1,46) = 5.31 *p* = 0.03	F(1,46) = 0.50 *p* = 0.48
*NOS2*	F(1,46) = 0.01 *p* = 0.94	F(1,46) = 4.09 *p* = 0.05	F(1,46) = 0.06 *p* = 0.80
*NR3C1*	F(1,46) = 0.67 *p* = 0.42	F(1,46) = 8.37 *p* = 0.01 0 < 1, *p* = 0.02 0 < 5, *p* = 0.003	F(1,46) = 0.03 *p* = 0.87
*SOCS1*	F(1,46) = 1.81 *p* = 0.19	F(1,46) = 5.91 *p* = 0.02	F(1,46) = 0.14 *p* = 0.71
*STAT3*	F(1,46) = 0.004 *p* = 0.95	F(1,46) = 5.11 *p* = 0.03	F(1,46) = 0.56 *p* = 0.46
*SYP*	F(1,46) = 0.89 *p* = 0.35	F(1,46) = 8.92 *p* = 0.005 0 < 1, *p* = 0.01 0 < 3, *p* = 0.02 0 < 5, *p* = 0.02	F(1,46) = 0.60 *p* = 0.81
*TMEM119*	F(1,46) = 0.03 *p* = 0.87	F(1,46) = 4.67 *p* = 0.04	F(1,46) = 3.26 *p* = 0.08
*TREM2*	F(1,46) = 2.53 *p* = 0.12	F(1,46) = 12.76 *p* < 0.001 0 < 1, *p* = 0.02 0 < 3, *p* = 0.03 0 < 5, *p* < 0.001	F(1,46) = 0.40 *p* = 0.53
*VCAM1*	F(1,46) = 0.25 *p* = 0.62	F(1,46) = 5.45 *p* = 0.02	F(1,46) = 1.95 *p* = 0.17
*VEGF-A*	F(1,46) = 0.003 *p* = 0.96	F(1,46) = 5.69 *p* = 0.02	F(1,46) = 0.74 *p* = 0.40

Red text denotes a significant effect (*p* < 0.01), and purple denotes those at *p* < 0.05). Sham mice are considered 0 injury

#### Gene expression was altered in the P21 HPC, but not PFC in AHT mice

RNA was also extracted from the HPC and PFC of mice at P21 for analysis of relative gene expression. A two-way ANOVA with sex and injury as factors was used. Significance was set at *p* < 0.01 to correct for multiple comparisons. In the HPC, 12/45 genes were significantly altered by injury; however, in the PFC there were no significant changes detected (Fig. 7C and D). It is worth noting that in the P21 HPC, there was an increase in gene expression of *AQP4*, and a gene involved in white matter production (*MYRF*) due to the AHT. Statistical analyses of the significantly affected genes are reported in Table 4.

**Table 4:** P21 PCR results following 60s injuries. Statistical results from the two-way ANOVA for gene expression in the P21 HPC and PFC following 1, 3, 5, or sham 60s injuries

Gene	ANOVA Results		
	Sex	Injury	Interaction
HPC			
*AQP4*	F(1,43) = 0.47 *p* = 0.50	F(1,43) = 8.90 *p* = 0.01 0 < 1, *p* = 0.003 0 < 3, *p* = 0.02	F(1,43) = 0.17 *p* = 0.68
*ARG1*	F(1,43) = 0.02 *p* = 0.90	F(1,43) = 5.61 *p* = 0.003 0 < 1, *p* = 0.002 1 < 3, *p* = 0.004 1 > 5, *p* = 0.001	F(1,43) = 0.23 *p* = 0.88
*BDNF*	F(1,43) = 0.04 *p* = 0.84	F(1,43) = 9.54 *p* = 0.004 0 > 1, *p* = 0.003 0 > 3, *p* < 0.001 3 < 5, *p* = 0.01	F(1,43) = 0.43 *p* = 0.52
*CRHR1*	F(1,43) = 4.25 *p* = 0.05 ♀<♂	F(1,43) = 0.29 *p* = 0.60	F(1,43) = 1.48 *p* = 0.23
*Eno2*	F(1,43) = 0.52 *p* = 0.48	F(1,43) = 4.09 *p* = 0.05	F(1,43) = 2.00 *p* = 0.17
*FOXP3*	F(1,43) = 0.14 *p* = 0.72	F(1,43) = 8.48 *p* = 0.01 0 > 1, *p* = 0.01 0 > 3, *p* = 0.01	F(1,43) = 0.02 *p* = 0.89
*GAP43*	F(1,43) = 1.13 *p* = 0.29	F(1,43) = 8.00 *p* = 0.003 0 < 1, *p* = 0.004 0 < 3, *p* = 0.001 3 > 5, *p* = 0.02	F(1,43) = 0.06 *p* = 0.80
*GHRH*	F(1,43) = 0.43 *p* = 0.52	F(1,43) = 7.36 *p* = 0.01 0 < 1, *p* = 0.004 0 < 3, *p* = 0.002 1 > 5, *p* = 0.02 3 > 5, *p* = 0.01	F(1,43) = 0.47 *p* = 0.50
*ICAM1*	F(1,43) = 0.99 *p* = 0.33	F(1,43) = 4.33 *p* = 0.04	F(1,43) = 0.04 *p* = 0.85
*IGF-1*	F(1,43) = 0.08 *p* = 0.78	F(1,43) = 6.63 *p* = 0.01 0 < 1, *p =* 0.02 0 < 3, *p =* 0.03	F(1,43) = 0.04 *p* = 0.85
*MAOA*	F(1,43) = 0.85 *p* = 0.36	F(1,43) = 4.51 *p* = 0.04	F(1,43) = 0.25 *p* = 0.62
*MapK*	F(1,43) = 0.04 *p* = 0.85	F(1,43) = 5.82 *p* = 0.02	F(1,43) = 0.14 *p* = 0.71
*MBP*	F(1,43) = 0.003 *p* = 0.96	F(1,43) = 4.43 *p* = 0.04	F(1,43) = 0.00 *p* = 1.00
*MYRF*	F(1,43) = 0.77 *p* = 0.39	F(1,43) = 13.19 *p* < 0.001 0 < 1, *p* < 0.001 0 < 3, *p* < 0.001 1 > 5, *p* = 0.03 3 > 5, *p* = 0.05	F(1,43) = 0.16 *p* = 0.67
*NES*	F(1,43) = 1.74 *p* = 0.19	F(1,43) = 7.32 *p* = 0.01 0 < 1, *p* = 0.03 0 < 3, *p* = 0.01	F(1,43) = 0.37 *p* = 0.55
*Nfkb1*	F(1,43) = 0.80 *p* = 0.34	F(1,43) = 6.85 *p* = 0.01 0 < 1, *p* = 0.01 0 < 3, *p* = 0.02	F(1,43) = 0.72 *p* = 0.40
*NOS2*	F(1,43) = 1.70 *p* = 0.20	F(1,43) = 11.22 *p* = 0.002 0 < 1, *p* = 0.004 0 < 3, *p* = 0.002 0 < 5, *p* = 0.05	F(1,43) = 0.15 *p* = 0.71
*NR3C2*	F(1,43) = 0.03 *p* = 0.86	F(1,43) = 7.21 *p* = 0.01 0 > 1, *p* = 0.01 0 > 3, *p* = 0.002 3 < 5, *p* = 0.02	F(1,43) = 0.27 *p* = 0.61
*PECAM1*	F(1,43) = 1.03 *p* = 0.32	F(1,43) = 4.58 *p* = 0.04	F(1,43) = 0.01 *p* = 0.92
*TMEM119*	F(1,43) = 1.26 *p* = 0.32	F(1,43) = 4.02 *p* = 0.05	F(1,43) = 1.34 *p* = 0.25

## Discussion

Paediatric AHT can manifest as a broad scope of deficits, ranging from mild lethargy to death [42]. Abusive behaviours, especially AHTs, are highly repetitive [2, 28, 65], which likely potentiates the damage produced from the injuries. Despite the severity and long-term impact of AHT, there are few animal models that accurately reflect the progression of deficits following repetitive mild injuries of this type. A robust preclinical model allows for the in-depth exploration of pathophysiological mechanisms underlying the consequences produced from AHTs. Prior preclinical models of AHT have used focal injuries, rotation on a single plane, or shaking devices similar to the model developed here [37]. Importantly, however, many previous models have used anaesthesia during the injury, confounding the results, and there is a paucity of research that has examined the long-term deficits produced from the model [37]. Therefore, the goal of this study was to develop a preclinical mouse model of AHT, that focused on replicating both brain pathology and the protracted manifestation of behavioural deficits, without the use of anaesthesia. Initially, a 3-injury, 15-second model was employed; however, this was insufficient to induce clinically relevant deficits, leading to the development of a 5-injury, 60-second model. This revised approach resulted in cumulative deficits due to repetitive injuries at acute, intermediate, and chronic stages. This study revealed evidence of brain oedema, altered gene expression, changes in white matter-forming cells, as well as the emergence of social deficits and altered nociception during adolescence, all resulting from repetitive AHT.

As previously noted, children with an AHT may initially present with no behavioural symptoms, only to develop deficits later in childhood and adolescence, years after the injury event [5, 6, 12, 13, 27, 49, 66]. In this current mouse study, no behavioural deficits were detected in the acute injury phase, which aligns with clinical observations in a proportion of children with AHT [5, 6, 12, 13, 27, 49, 66]. It is possible that the developmental behaviours assessed with our protocols early after early-life injury were highly evolutionarily conserved, rudimentary, and reflexive, and as such were not altered in response to repeat mild injuries. Furthermore, the early neonatal brain is highly plastic, with built-in redundancy [39, 99], which may allow for compensation after milder injuries [96]. However, this capacity to compensate depends on the location and timing of injury, with deficits becoming more pronounced with time post-injury [51, 96, 99].

In this study, while neonatal behaviours were unaltered, adolescent mice exhibited changes to social behaviour due to injury, particularly displaying an increase in time spent following. Social behaviour is highly complex and given that we were only able to examine one social task, the results must be interpreted with caution. However, the altered social following produced in these animals, still provides valuable information regarding the social deficits associated with AHT. Following behaviour is typically affiliative and investigative [30, 103], and the increased duration depicted here may indicate that AHT mice needed more time to gather the same social information as their uninjured counterparts. A study examining paediatric moderate-severe TBI induced at P21 produced no significant differences in social interaction during adolescence, but when tested in adulthood, following behaviour and sniffing were decreased, and aggression was increased in injured mice [88]. This further emphasises the importance of injury timing and the developmental window of assessment in behavioural outcomes. While there are many genetic, epigenetic, and environmental factors that contribute to socialisation, we noted that the gene *MAOA* was altered in the hippocampus of injured mice at the acute timepoint and a trend for increased expression at the intermediate time point. As *MAOA* has been linked to antisocial personality disorder and aggression [4, 54], it is possible that this change may be linked to the social deficits produced in this model. However, further tests are required to examine if this link is causal or if *MAOA* changes of this magnitude would result in increased aggression in adulthood. Further, *MAOA* was not altered in the PFC, a brain region instrumental for social behaviour [90], and it is possible that is why we only identified minimal social dysfunction. Additionally, social behaviour is vital to survival and there is likely redundancy in genes involved in social behaviour. For example, *IGF-1* was increased in the PFC at the acute timepoint in this model, and increased *IGF-1* expression has been linked to the rewarding aspects of play behaviour [90], and this may result in the increased social following identified here. Paediatric TBIs alter the dopaminergic reward pathways in the brain and increase the risk of addiction [15]. Adolescence is a time period associated with increased sensitivity to reward, and given that following in mice is a prosocial behaviour, the early life abuse may have altered the reward pathways, subsequently leading to increased sensitivity to the rewarding aspects of affiliative following behaviour [25, 38, 43].

Regarding pain sensitivity, responsiveness to thermal nociception was increased in AHT mice, accompanied by alterations in genes involved in nociceptive processing, such as *MapK* [47], *Egr-1* [50], and *IGF-1* [70]. While, to our knowledge, the development of chronic pain has not been specifically examined in children with an AHT, TBI in general has been linked to an increased prevalence of chronic pain [21, 56, 74, 86]. These results suggest that AHT may influence pain perception long after the injury occurred. Finally, while no significant changes in anxiety-like behaviours were observed in this study, several genes associated with anxiety, such as *CRHR1* [95], *NR3C1* [101], *NR3C2* [14], were altered in a region and time dependent manner injured mice. Increased vulnerability to the development of anxiety is observed in children with an AHT [3, 60], and it is possible that the developmental timing of the behavioural test in this study was not optimal for detecting changes in anxiety-like behaviour. Further studies are required to determine if alterations in anxiety-like behaviours are manifested in this model at later time-points.

Alterations in inflammatory, immune, and white matter cell quantities at acute and intermediate time points were examined as potential markers of brain pathology. Specifically, the HPC was a focal point for analysis as it is highly conserved [22], and has a heightened vulnerability to injury [24, 64, 69]. Additionally, we examined the PFC, as this is one of the last brain regions to develop [33, 53], and continues to undergo proliferation and maturation across the time period that AHTs typically occur [37]. There were no observable effects following AHT in inflammatory or myeloid immune cells in the HPC or PFC as assessed by astrocyte and microglial staining, respectively. One possible explanation is that the whiplash mechanism of injury, characteristic of AHT, lacks a specific focal point of damage [19, 46, 78], and therefore, other brain regions not currently examined may have been diffusely affected. Alternatively, the timing of the assessments might not have coincided with peak immune activation, particularly in response to repetitive, mild injury. For instance, another study indicated that significant increases in microglial cell number following a TBI were apparent 28 days following injury, despite being unaltered 24 h after injury [16]. Considering that neurogenesis, migration, and pruning are ongoing processes that occur in the neonatal period, cell birth and death were also examined. Again, no significant differences were identified in AHT mice compared to sham mice. Due to the dynamic nature of cell turnover during this period, the developing brain may be able to compensate for these injuries. Moreover, there is a “sensitive period” in early brain development, during which the brain and specific cell types are especially vulnerable to injury, with recovery being more robust outside of this period [52].

White matter is highly susceptible to shear and strain injuries that commonly occur from whiplash injuries, such as AHT [1, 41, 94]. We identified an increase in oligodendrocytes, the primary cells responsible for myelin formation within the HPC. Furthermore, the expression of *MYRF*, a gene involved in myelin formation [59], was significantly elevated in the PFC at P14 and the HPC at P21 following AHT. These findings indicate that diffuse shearing injuries may trigger compensatory mechanisms, resulting in an upregulation of white matter formation. Interestingly, white matter upregulation was specific to the HPC and PFC and was not observed in the major white matter tract of the brain, the corpus callosum. This further underscores the specific vulnerabilities of the PFC and HPC to AHTs during a critical period of neonatal development.

Subdural haemorrhages leading to oedema and increased intracranial pressure is the primary cause of death in children with an AHT [35]. Although mortality was not observed in this model, significant oedema and breakdown of the BBB was evident, particularly following 5 AHTs. Preclinical models of TBI’s indicate that increased AQP4 channels and increased brain weight are indicative and correlate to brain oedema [32, 72, 97]. Increased brain weight was only detected 48 h following AHTs, but this had returned to baseline by one week. However, increased BBB leakage persisted for one week following AHT. Furthermore, the BBB dysfunction produced in this model was accompanied by an increase in AQP4 channel immunofluorescence in the HPC, and in the PFC of females. The changes in AQP4 immunofluorescence in the HPC was complimented by increased gene expression at P21, that was not apparent in the PFC. AQP4 channels are instrumental in maintaining brain water homeostasis [44], cerebrospinal fluid production [20, 44], and waste clearance from the brain parenchyma [45]. Interestingly, reducing AQP4 in a model of paediatric TBI not only decreased oedema and BBB dysfunction, but also improved acute and chronic behavioural deficits [31]. While these results may indicate altering AQP4 could be a promising therapeutic following an AHT, the timing and how to specifically target the HPC must be considered when designing any pharmacological therapies. Although AQP4 may cause cerebral oedema, it is also responsible for oedema clearance, and therefore chronic depletion may result in broad detrimental outcomes for recovery and healthy development.

Both sexes were included in this study, but overall, there were very few parameters that were differentially affected by sex. As expected, males gained more weight than females throughout the duration of the study, regardless of injury status. Males also had increased brain weight at P14 compared to females and had increased expression of *Egr-1* in the P14 PFC. *Egr-1* is involved in cell proliferation and cell growth [8], and the increased expression in males may be related to the increased brain weight and growth early in life.

Expression levels of 45 different genes were examined in the HPC and PFC at acute and intermediate time points to assess AHT-induced damage. The P14 PFC demonstrated the greatest injury-induced change to gene expression, with every altered gene exhibiting increased expression following the AHTs. It is interesting to note that at P21, AHT-induced changes to PFC gene expression had normalised. These results indicate that the ongoing development and growth during this time may have allowed the PFC to compensate for the injuries. Conversely, the HPC displayed different trends, with the P14 HPC exhibiting less change in gene expression when compared to the P21 HPC. In addition, when genes in HPC were altered at both time points, they unexpectedly displayed opposite changes to expression levels at P14 and P21. For example, genes involved in growth such as *GHRH* and *IGF-1*, exhibited decreased expression from injury at the acute timepoint, but subsequently had increased expression at P21. The HPC and PFC are at different developmental stages when the AHTs occurred and therefore respond and recover differently to damage [51, 52], hence we hypothesise that the PFC may have been able to compensate, while the HPC was unable to.

Only three genes were unaffected across all time points and brain regions analysed. One of those unaffected genes, *TNFα*, is of particular interest due to its broad use as a marker of inflammation including in concussion [80, 93]. However, expression levels of serum *TNFα* generally peak within hours after injury [23, 29], and the earliest timepoint we examined was 48 h post-injury, suggesting that we had missed the potential peak in expression. Interestingly, *NfL* is commonly upregulated in the serum of adults following a concussion [26, 68, 91], but in our model *NfL* was decreased by injury only in the P14 HPC. However, it may not be appropriate to compare *NfL* expression in the neonatal brain to expression in adult serum. *NfL* is a structural protein involved in axon stability and generation [34], but another protein involved in neurogenesis and axon guidance, *EfnB* [82], was increased in the same region and timepoint. Taken together, this suggests that although increased *NfL* in serum following injury in adults may indicate damage, in the neonatal brain where axon growth is more prominent, expression of genes involved in neurogenesis may be more indicative of trauma. Nevertheless, expression of genes that indicate cell damage were acutely upregulated in the PFC of injured mice, including *SYP* [36, 63], *Eno2* [9], and *Hif-1α* [58].

While this study aimed to develop a preclinical model of AHT, there are a few important limitations to discuss. Namely, not all of the features that manifest in human populations who experience AHT were replicated, such as lethargy. Additionally, this model represents a mild injury, where increasing the number of injuries should culminate in increased pathology, however this was not the case for all molecular, immunohistochemistry, and behavioural tests employed. For example, while AQP4 fluorescence increased in a cumulative manner with each successive injury, Olig2 cell counts were only modified in response to single and 3 injuries, but was not evident following 5 injuries. As with human children, there is variability in the manifestation of pathology, and further exploration needs to be done to tease apart the important clinical manifestations of repetitive injuries and how different measures may respond in a dose dependent manner. For instance, some pathologies may be protected by a prior injury, as indicated in another study whereby a precisely timed mTBI protected the brain from a later mTBI [73]. To contrast this, some preclinical TBI models identify similar outcomes from single and repetitive injuries [76], while others demonstrate exacerbation of outcomes in the context of repeat injuries [10].

## Conclusion

In summary, this study developed a novel preclinical neonatal mouse model of AHT that successfully replicated key clinical features observed in human cases. This model demonstrated the progression of deficits following single and repetitive injuries, highlighting the cumulative effects of repetitive AHTs on behaviour and brain pathology. Notably, to our knowledge, this is the first animal model where acute behavioural deficits were initially absent, but emerged during adolescence, closely mirroring human developmental patterns. Furthermore, this model produced alterations in nociception which is a potentially unrecognised issue that requires further examination in human AHT survivors. Although the behavioural deficits required time to manifest, we observed acute alterations in white matter cells and gene expression in a region and time dependent manner. This finding opens the door for the exploration of potential treatments that can be administered to attenuate the onset of behavioural deficits that emerge later in life. Additionally, the changes in gene expression identified in this model could inform targeted research focussed on restoring these changes to support healthy development of children affected by AHT. We also identified increased BBB leakage from repetitive injuries, but not from a single injury. This is significant, as increased intracranial pressure is the leading cause of mortality in human children [35], and monitoring BBB permeability, for example via non-invasive magnetic resonance imaging techniques [61, 62], could enable earlier intervention to help identify instances of prior AHT. Although preclinical models are unable to examine more complex behaviours, such as language development, implementing advanced and sensitive behavioural tasks could provide further insight to the deficits produced by AHT. Overall, the development of this preclinical model provides the research field with a valuable tool for the exploration of diagnostic biomarkers and therapeutic interventions for children affected by AHT.

## Electronic supplementary material

Below is the link to the electronic supplementary material.

Supplementary Material 1

## Data Availability

All raw data will be made available at the open-source framework site https://osf.io/2kg5j/?view_only=945c457a3bdd4cb88294f04fdbd461c0 upon publication.
