# Supplementary material for: Shaking into deficits: investigating behavioural and neuropathological outcomes associated with a novel preclinical model of infant abusive head trauma
Source: Acta Neuropathol Commun. 2025 May 15;13:100. doi: 10.1186/s40478-025-02029-5 (PMC12079959; doi:10.1186/s40478-025-02029-5)
Supplement: Supplementary file 1 — Supplementary Material 1 [file 40478_2025_2029_MOESM1_ESM.docx]

Supplementary Information:

**Supplementary Table 1:** Genes analysed in High Throughput Fluidigm qPCR.

| Gene | Name | Taqman Assay ID |
| --- | --- | --- |
| *Apaf-1* | Apoptotic peptidase activating factor 1 | Mm01223702_m1 |
| *AQP4* | Aquaporin4 | Mm00802131_m1 |
| *ARG1* | Arginase1 | Mm00475988_m1 |
| *BDNF* | Brain derived neurotrophic factor | Mm04230607_s1 |
| *CCL2* | Monocyte chemoattractant | Mm00441242_m1 |
| *CNR1* | Cannabinoid receptor 1 | Mm01212171_s1 |
| *CRHR1* | Corticotropin releasing hormone receptor 1 | Mm00432670_m1 |
| *EfnB* | EphrinB2 | Mm00438670_m1 |
| *Egr-1* | Early growth response protein | Mm00656724_m1 |
| *Eno2* | Neuron specific enolase | Mm01185009_gH |
| *FOXP3* | Forkhead box P3 | Mm00475162_m1 |
| *GABRA1* | Gaba receptor subunit 1 | Mm00439046_m1 |
| *GAP43* | Growth associated protein 43 | Mm00500404_m1 |
| *GATA3* | GATA Binding protein | Mm00484683_m1 |
| *GFAP* | Glial fibrillary acidic protein | Mm01253033_m1 |
| *GHRH* | Growth hormone releasing hormone | Mm00439100_m1 |
| *GSK3B* | Glycogen synthase kinase 3-beta | Mm00444911_m1 |
| *HIF-1a* | Hypoxia inducible factor 1 | Mm00468869_m1 |
| ***Hprt*** | Hprt | Mm03024075_m1 |
| *HSPA9* | Heat shock protein family A | Mm00477716_g1 |
| *Iba1* | Allograft inflammatory marker 1 | Mm00479862_g1 |
| *ICAM1* | Intracellular adhesion molecule | Mm00516023_m1 |
| *IGF-1* | Insulin like growth factor | Mm00439560_m1 |
| *IL-1*$\beta$ | Interleukin 1 beta | Mm00434228_m1 |
| *IL-6* | Interleukin6 | Mm00446190_m1 |
| *MAOA* | Monoamine oxidase A | Mm00558004_m1 |
| *MapK* | Mitogen activated protein kinase A | Mm00442479_m1 |
| *MBP* | Myelin basic protein | Mm01266402_m1 |
| *MYRF* | Myelin regulatory protein | Mm01194959_m1 |
| *NES* | Nestin | Mm00450205_m1 |
| *NF-kB* | Nuclear factor kappa B | Mm00476361_m1 |
| *NfL* | Neurofilament light protein | Mm01315666_m1 |
| *NLRP3* | NLR Family Pyrin Domain Containing 3 | Mm00840904_m1 |
| *NOS2* | Nitric oxide synthase 2 | Mm00440485_m1 |
| *NR3C1* | Glucocorticoid receptor | Mm00433832_m1 |
| *NR3C2* | Mineralcorticoid receptor | Mm01241596_m1 |
| *PECAM-1* | Platelet endothelial cell adhesion molecule | Mm01242576_m1 |
| *PLVAP* | Plasmalemmal vesicle associated protein-1 | Mm00453381_g1 |
| ***Ppia*** | Ppia | Mm02342430_g1 |
| *SOCS1* | Suppressor of cytokine signalling 1 | Mm00782550_s1 |
| *STAT3* | Signal transducer and activator of transcription | Mm01219775_m1 |
| *SYP* | Synaptophysin | Mm00436850_m1 |
| *TMEM119* | Transmembrane protein 119 | Mm00525305_m1 |
| *TNF*$\alpha$ | Tumor necrosis factor | Mm99999068_m1 |
| *TREM2* | Triggering receptor expressed on myeloid cells 2 | Mm04209424_g1 |
| *VCAM1* | Vascular cell adhesion molecule | Mm01320970_m1 |
| *VEGF-A* | Vascular Endothelial Growth Factor A | Mm00437304_m1 |
| ***Ywhaz*** | Ywhaz | Mm01722325_m1 |

Genes are listed in alphabetical order, and housekeeping genes are bolded.

**Supplementary Table 2**: Immunohistochemistry antibodies, concentrations and regions imaged and analysed.

| Primary Ab (dilution) Company, Cat # | Secondary Ab  (dilution), species  Company, Cat # | Image frequency | Region and # sections analysed |
| --- | --- | --- | --- |
| GFAP (1:1000)  Anti-Glial Fibrillary Acidic Protein clone GA5 mouse monoclonal,  Millipore, MAB360 | AlexaFluor594 (1:500)  Donkey anti-mouse  Invitrogen, A21203 | 1 image every 700$\mu$m | HPC  3 sections |
|  |  | 1 image every 420$\mu$m | mPFC and lPFC  4 sections |
| Olig2 (1:500)  Rabbit anti-Olig2 Polyclonal,  Millipore, AB9610 | AlexaFluor488 (1:500)  Donkey anti-rabbit  Invitrogen, A21206 | 1 image every 700$\mu$m | HPC  3 sections |
|  |  | 1 image every 420$\mu$m | Anterior CC  3 sections |
| Iba1 (1:1000)  Goat anti-Iba1  Polyclonal  Abcam ab5076 | AlexaFluor647 (1:500)  Donkey anti-goat  Abcam, ab150131 | 1 image every 420$\mu$m | mPFC and lPFC  4 sections |
|  |  | 1 image every 700$\mu$m | HPC  3 sections |
| Ib4 (1:250)  Conjugated to FITC  Lectin from *Bandeirara simplicifolia*  Sigma-Aldrich L2895 | N/A | 1 image every 700 $\mu$m | Whole brain  9 sections |
| AQP4 (1:1000)  Rabbit anti-AQP4  Monoclonal Abcam ab128906 | AlexaFluor488 (1:500)  Donkey anti-rabbit  Invitrogen, A21206 | 1 image every 420$\mu$m | mPFC and lPFC  4 sections |
|  |  | 1 image every 700$\mu$m | HPC  3 Sections |
| Ki67 (1:250)  Rabbit anti-Ki67  Monoclonal Invitrogen  MA514520 | AlexaFluor488 (1:500)  Donkey anti-rabbit  Invitrogen, A21206 | 1 image every 700$\mu$m | HPC  3 sections |
| SOCS1 (1:500)  Mouse anti-SOCS1 clone4H1  Monoclonal Invitrogen AB2533316 | AlexaFluor594 (1:500)  Donkey anti-mouse  Invitrogen, A21203 | 1 image every 700$\mu$m | HPC  3 sections |

**Supplementary Table 3**: Medial prefrontal cortex (mPFC) and lateral prefrontal cortex (lPFC) region of interest delineations for each brain section.

| Region | Anterior position relative to Bregma | Dorsal position | Lateral Position | Height | Width |
| --- | --- | --- | --- | --- | --- |
| mPFC | +2.10 | -500 $\mu$m | Midline | 500 $\mu$m | 1000 $\mu$m |
|  | +1.70 | -750 $\mu$m | Midline | 750 $\mu$m | 1000 $\mu$m |
|  | +1.30 | -1000 $\mu$m | Midline | 500 $\mu$m | 1500 $\mu$m |
|  | +0.80 | -500 $\mu$m | Midline | 1000 $\mu$m | 1500 $\mu$m |
| lPFC | +2.10 | -1750 $\mu$m | Outermost periphery | 750 $\mu$m | 750 $\mu$m |
|  | +1.70 | -2250 $\mu$m | Outermost periphery | 750 $\mu$m | 750 $\mu$m |
|  | +1.30 | -2250 $\mu$m | Outermost periphery | 500 $\mu$m | 1000 $\mu$m |
|  | +0.80 | -3000 $\mu$m | Outermost periphery | 750 $\mu$m | 1000 $\mu$m |
